# Evolutionary mechanisms that determine which bacterial genes are carried on plasmids

**DOI:** 10.1002/evl3.226

**Published:** 2021-05-18

**Authors:** Sonja Lehtinen, Jana S. Huisman, Sebastian Bonhoeffer

**Affiliations:** ^1^ Department of Environmental System Science Institute for Integrative Biology, ETH Zürich Universitätstrasse 16 Zürich 8006 Switzerland; ^2^ Swiss Institute of Bioinformatics Quartier Sorge Lausanne 1015 Switzerland

**Keywords:** Evolutionary genomics, microbes, models, theory

## Abstract

The evolutionary pressures that determine the location (chromosomal or plasmid‐borne) of bacterial genes are not fully understood. We investigate these pressures through mathematical modeling in the context of antibiotic resistance, which is often found on plasmids. Our central finding is that gene location is under positive frequency‐dependent selection: the higher the frequency of one form of resistance compared to the other, the higher its relative fitness. This can keep moderately beneficial genes on plasmids, despite occasional plasmid loss. For these genes, positive frequency dependence leads to a priority effect: whichever form is acquired first—through either mutation or horizontal gene transfer—has time to increase in frequency and thus becomes difficult to displace. Higher rates of horizontal transfer of plasmid‐borne than chromosomal genes therefore predict moderately beneficial genes will be found on plasmids. Gene flow between plasmid and chromosome allows chromosomal forms to arise, but positive frequency‐dependent selection prevents these from establishing. Further modeling shows that this effect is particularly pronounced when genes are shared across a large number of species, suggesting that antibiotic resistance genes are often found on plasmids because they are moderately beneficial across many species. We also revisit previous theoretical work—relating to the role of local adaptation in explaining gene location and to plasmid persistence—in light of our findings.

**Impact Summary**

Bacterial genes can either reside on the chromosome or on plasmids, extrachromosomal genetic structures, which can be transferred from cell to cell. The distribution of genes between plasmid and chromosome is not random: certain types of genes are particularly likely to be plasmid‐associated. This includes a number of clinically important traits, such as antibiotic resistance and virulence factors. The evolutionary mechanisms that give rise to this pattern are not well understood. Plasmids are occasionally lost during cell replication and thus less reliably inherited than the chromosome, and genes are free to transition between plasmid and chromosome: so what keeps genes on plasmids? We address this question using a mathematical model. The key prediction from our model is that the relative fitness of chromosomal and plasmid‐borne genes depends on their relative frequencies (positive frequency‐dependent selection). In other words, the fitness of a plasmid‐borne gene will be higher in a population in which the chromosomal gene is rare (and vice versa). This positive frequency dependence can keep moderately beneficial genes on plasmids, despite occasional plasmid loss. This leads to a priority effect: whichever form of the gene (i.e., plasmid‐borne or chromosomal) is acquired first has time to increase in frequency and thus becomes difficult to displace. Therefore, the relative rate of acquiring the gene on the plasmid versus the chromosome predicts where the gene will be found. Further modeling shows this effect is particularly pronounced when genes are beneficial across a large number of species. All together, the hypothesis that emerges from our work is that plasmid‐borne genes are moderately beneficial; functional across a large number of species; and rarely acquired through chromosomal mutation. We suggest traits like antibiotic resistance are often found on plasmids because these genes commonly fulfill these criteria.

Plasmids are extra‐chromosomal genetic structures that can replicate independently from the chromosome and be transferred from cell to cell. These structures play a key role in bacterial evolution: in addition to genes that enable their own replication and spread, plasmids can carry genes that are beneficial to the bacterial host. This makes plasmids an important vehicle of horizontal gene transfer, both within and between species.

There is evidence that the types of genes found on plasmids differ from those found on the chromosome. In general terms, plasmid‐borne genes are part of the accessory rather than core genome (Hacker and Carniel 2001; Rankin et al. 2011). Furthermore, some accessory functions appear to be particularly likely to be plasmid associated (Eberhard 1989; Rankin et al. 2011; Carroll and Wong 2018)—these include virulence factors (Johnson and Nolan 2019), antibiotic resistance (Svara and Rankin 2011; Rozwandowicz et al. 2018), heavy metal tolerance Piotrowska‐Seget et al. 2005), and bacteriocins (toxins involved in interstrain competition; Riley and Wertz 2002).

These patterns are intriguing: genes can transit between plasmids and the chromosome (Sheppard et al. 2016); thus, over evolutionary time‐scales, genes are likely to have experienced both plasmid and chromosomal backgrounds (Eberhard 1989). There must therefore be evolutionary mechanisms that give rise to the association between plasmids and particular gene functions. Understanding these mechanisms is important, both because of the role plasmids play in bacterial evolution and because of the clinical relevance of plasmid‐associated functions.

The absence of core genes from plasmids is relatively well understood. First, core genes are more likely to be highly connected in cellular networks (Jeong et al. 2001), thus making them less transferable across genetic backgrounds (complexity hypothesis; Cohen et al. 2011). Second, inheritance of plasmids is less stable than inheritance of chromosomes: daughter cells do not always inherit a copy of the plasmid during cell division (segregation loss). Thus, if a gene is essential to the survival of the cell, we would expect it to locate onto the chromosome. Plasmids encode mechanisms to prevent segregation loss (Zielenkiewicz and Cegłowski 2001) and plasmid loss is therefore rare (Lau et al. 2013), but even low rates of plasmid loss are predicted to make essential genes chromosomal rather than plasmid‐borne (Tazzyman and Bonhoeffer 2015). Third, horizontal gene transfer can allow lineages to recover lost genes (Bergstrom et al. 2000; Vogan and Higgs 2011; Takeuchi et al. 2014), but this mechanism is less relevant to essential genes, as lineages without these genes would themselves be lost (Hall et al. 2017).

The overrepresentation of particular functions on plasmids remains puzzling. In the specific case of cooperative traits, one possible explanation is that mobility is beneficial because it enforces the cooperation of neighboring cells (Smith 2001; Nogueira et al. 2009; Rankin et al. 2011). More generally, it has been suggested that plasmid‐borne genes code for local adaptation: if a gene is beneficial in a specific environment, being plasmid‐borne would allow this gene to spread into immigrant lineages lacking the trait, thus maintaining the gene on the plasmid (Eberhard 1989; Bergstrom et al. 2000). A related suggestion is that temporal fluctuation in selection pressure would favor plasmid‐borne genes: being plasmid‐borne would allow the gene to increase in frequency faster during periods of positive selection and to persist through plasmid transfer during period of negative selection (Svara and Rankin 2011; Stevenson et al. 2018).

The local adaptation explanation is based on the assumption that the immigrant lineage is susceptible to infection by the local plasmid, that is, it does not already carry the plasmid (either with or without the locally beneficial gene). This assumption arises from estimates of plasmid transfer rates in liquid culture, which suggest that plasmids are not transmissible enough, relative to their cost and the rate of plasmid loss, to exists as pure parasites (i.e., without carrying genes beneficial for the host). Thus, outside the local niche with the beneficial gene, plasmids would not persist (Bergstrom et al. 2000).

More recently however, this assumption has been called into question (Lili et al. 2007). Estimates of plasmid transfer rates are higher in biofilms than in liquid culture (Sørensen et al. 2005) and there is evidence that compensatory evolution acts to ameliorate plasmid cost (San Millan et al. 2014; Harrison et al. 2015), particularly in the presence of spatially heterogeneous selection (Harrison et al. 2018). Indeed, recent experimental evidence suggests conjugative plasmids can persist through plasmid transfer despite not being beneficial (Lopatkin et al. 2017). Furthermore, genomic studies detect the same plasmid backbones with different gene content (e.g. with and without particular resistance genes; Sheppard et al. 2016). Taken together, this evidence suggests models of plasmid dynamics should include the possibility of both gene‐bearing and gene‐free versions of the plasmid.

We therefore revisit the question of why some genes are carried on plasmids, accounting for competition between plasmids, as well as chromosomes, with and without the focal gene. This yields a relatively complex model, which is difficult to study analytically (though previous work has addressed such a model through simulation; Svara and Rankin 2011). Here, we adopt an approach that makes the otherwise analytically unsolvable model mathematically tractable. For readability, our model is framed in terms of antibiotic resistance genes, though our results are more broadly applicable. Through a combination of analysis and simulation, we find that, indeed, in presence of the nonbeneficial plasmid, local adaptation does not explain why some genes are plasmid‐borne. However, gene location is under positive frequency‐dependent selection, which leads to a priority effect: whichever form is acquired first has time to increase in frequency and thus become difficult to displace. The presence of resistance genes on plasmids could therefore be explained by a greater rate of acquisition of plasmid‐borne than chromosomal resistance.

## Results

### MODEL

We consider a population consisting of cells which can be either chromosomally resistant or chromosomally sensitive (R or S), with no plasmid, a resistant plasmid, or a sensitive plasmid (∅, R, or S). This gives rise to six possible cell types: R∅, RR, RS, S∅, SR, and SS (the first letter denotes the chromosome, the second letter the plasmid).

**Figure 1:**
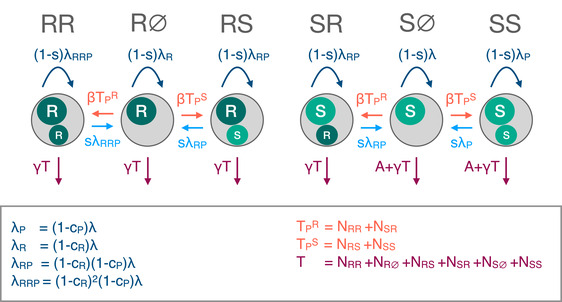
Schematic of the modeled dynamics (eq. 1). Each grey circle represents a cell type, with the interior circles representing the chromosome (large) and plasmid (small), with R denoting resistance and S sensitivity. The arrows indicate modeled processes: cell replication (dark blue), death (purple), plasmid transmission (orange), and segregation loss (light blue). The labels indicate the rate at which these processes occur. N indicates the density of a cell type, so T is the total cell density ($T=N_{S\emptyset }+N_{SS}+N_{SR}+N_{R\emptyset }+N_{RS}+N_{RR}$), $T_{P^{R}}$ is the total density of cells with a resistant plasmid ($T_{P^{R}}=N_{SR}+N_{RR}$), and $T_{P^{S}}$ is the total density of cells with a sensitive plasmid ($T_{P^{S}}=N_{SS}+N_{RS}$). λ is the replication rate; γ the density dependent death rate; A the antibiotic‐associated death rate; β the plasmid transmission rate; s the probability of segregation loss; $c_{P}$ the cost of plasmid carriage and $c_{R}$ the cost of carrying the resistance gene.

We begin by developing a model of the dynamics of these cells (Fig. 1) and denote the density of each cell type by $N_{•}$. Cells replicate at rate λ. Competition between cells is captured through a density‐dependent death rate γT, where T is the total cell density ($T=N_{S\emptyset }+N_{SS}+N_{SR}+N_{R\emptyset }+N_{RS}+N_{RR}$). Plasmids spread through density‐dependent transmission between cells at rate β and are lost during cell replication with probability s (segregation loss). Plasmid carriage is associated with a fitness cost $c_{P}$, which reduces the replication rate λ by a factor of $1-c_{P}$. We assume that cells can only be infected with one plasmid at a time. Cells with no resistance (S∅ and SS) experience an additional death rate A from antibiotic exposure. Resistance is associated with a fitness cost, which reduces the replication rate by a factor of $1-c_{R}$. We assume that resistance genes have the same fitness cost and the same effectiveness whether they are chromosomal or plasmid‐borne. Cells that have both chromosomal and plasmid‐borne resistance experience a dual fitness cost ${\left(1-c_{R}\right)}^{2}$. The effect of modifying these assumptions is explored in the Supporting Information (Section 2). Our main results are generally robust, with sensitivities highlighted in the main text.

The arising dynamics are described by the following equations:

(1) $$\begin{array}{ccc} \frac{dN_{S\emptyset }}{dt} & = & N_{S\emptyset }\left[\lambda -\beta \left(N_{SS}+N_{SR}+N_{RS}+N_{RR}\right)-\gamma T-A\right]\\ & & +\;s(\lambda_{P}N_{SS}+\lambda_{RP}N_{SR})\\ \frac{dN_{SS}}{dt} & = & N_{SS}\left[\left(1-s\right)\lambda_{P}-\gamma T-A\right]+\beta \left(N_{SS}+N_{RS}\right)N_{S\emptyset }\\ \frac{dN_{SR}}{dt} & = & N_{SR}\left[\left(1-s\right)\lambda_{RP}-\gamma T\right]+\beta \left(N_{SR}+N_{RR}\right)N_{S\emptyset }\\ \frac{dN_{R\emptyset }}{dt} & = & N_{R\emptyset }\left[\lambda_{R}-\beta \left(N_{SS}+N_{SR}+N_{RS}+N_{RR}\right)-\gamma T\right]\\ & & +\;s(\lambda_{RP}N_{RS}+\lambda_{RRP}N_{RR})\\ \frac{dN_{RS}}{dt} & = & N_{RS}\left[\left(1-s\right)\lambda_{RP}-\gamma T\right]+\beta \left(N_{SS}+N_{RS}\right)N_{R\emptyset }\\ \frac{dN_{RR}}{dt} & = & N_{RR}\left[\left(1-s\right)\lambda_{RRP}-\gamma T\right]+\beta \left(N_{SR}+N_{RR}\right)N_{R\emptyset }, \end{array}$$

with

$$\begin{array}{ccc} \lambda_{R} & = & (1-c_{R})\lambda \\ \lambda_{P} & = & (1-c_{P})\lambda \\ \lambda_{RP} & = & \left(1-c_{R}\right)\left(1-c_{P}\right)\lambda \\ \lambda_{RRP} & = & {\left(1-c_{R}\right)}^{2}\left(1-c_{P}\right)\lambda . \end{array}$$

The model parameters are summarized in Table 1. We expect parameter values (i.e., rates and costs) to differ considerably depending on, for example, bacterial species, type of plasmid, antibiotic, and environment. Our aim is to understand the behavior of a generalized system qualitatively, rather than make quantitative predictions about a specific system. We therefore explore a wide range of parameter values (Supporting Information Section 1) rather than choosing parameters to reflect a particular system.

**Table 1:** List of parameters, with their dimensions, the value used in the main text, ranges explored in Supporting Information Section 1, and the effect on evolutionary outcome

Parameter	Definition	Dimensions	Main text value (SI range)	Bistability when
λ	Replication rate	Time^−1^	1 (0.5, 2)	High
γ	Death rate	Volume cells^−1^ time^−1^	1 (0.5, 2)	Low
A	Antibiotic‐associated death rate	Time^−1^	1 (0, 2)	Low
$c_{R}$	Cost of antibiotic resistance	Dimensionless	0.05 (0, 0.5)	High
$c_{P}$	Cost of plasmid carriage	Dimensionless	0.075 (0, 0.5)	Low
β	Plasmid transmission rate	Volume cells^−1^ time^−1^	0.2 (0, 0.25)	High
s	Segregation loss	Dimensionless	0.005 (0, 0.1)	Low

*Note*: More specifically, the fifth column indicates whether the region of bistability (where resistance can be either plasmid‐borne or chromosomal) occurs at high or low parameter values compared to the region where only chromosomal resistance is evolutionarily stable (see Supporting Information Section 1). The parameter units are arbitrary. The main text values are chosen to best illustrate the range of evolutionary stable outcomes.

### EVOLUTIONARY STABILITY OF PLASMID‐BORNE AND CHROMOSOMAL RESISTANCE

**Figure 2:**
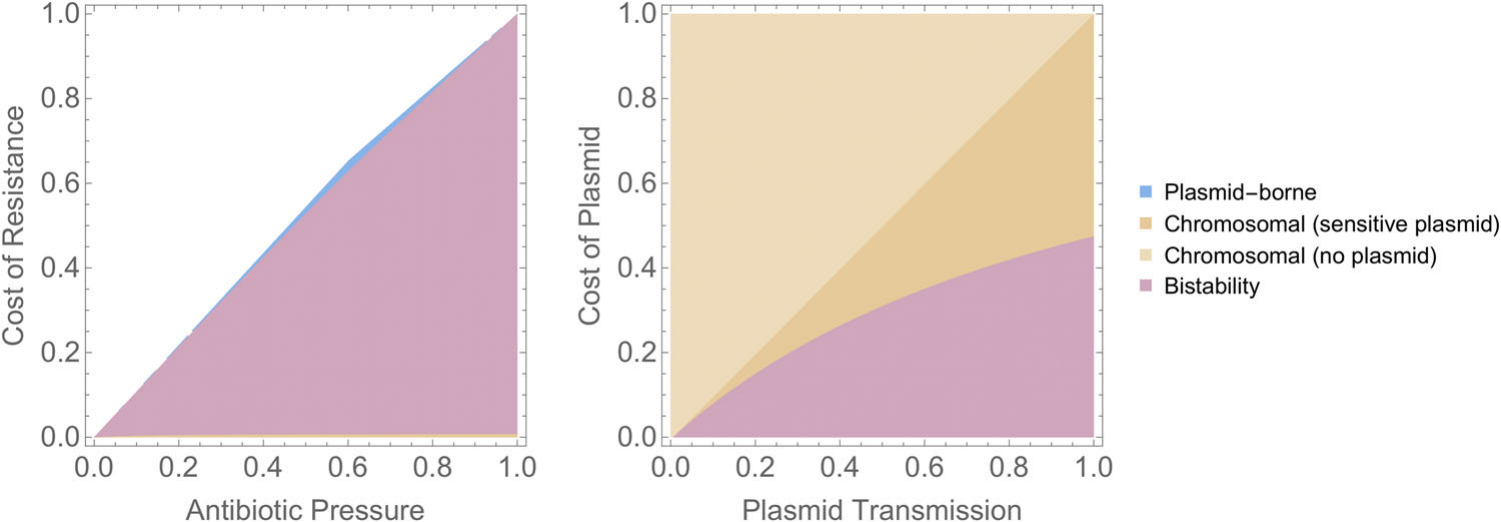
Evolutionary stability of plasmid‐borne and chromosomal resistance. The colors indicate which form of resistance is evolutionarily stable: only chromosomal resistance (orange); only plasmid‐borne resistance (blue); or either (purple). When resistance is chromosomal, the sensitive plasmid can either be present or absent from the population (dark vs light orange). In the white space in the left‐hand panel, neither form of resistance is stable (the population is antibiotic‐sensitive). Parameter values are: λ=1, γ=1, s=0.005. For left‐hand panel $c_{P}$ = 0.075 and β=0.2. For right‐hand panel A=1 and $c_{R}=0.05$. Figures S1 and S2 show results for more parametrizations. Note that in the parameter space where resistance is beneficial, it can either be essential (A≥λ: antibiotic susceptible cells are not viable, even in the absence of competition from resistant cells) or non‐essential. This distinction does not impact our results (Figs. S1 and S2).

We are interested in the evolutionary stability of chromosomal and plasmid‐borne resistance, that is, whether established chromosomal resistance can be displaced by plasmid‐borne resistance and vice versa. We determine parameter regions in which each type of resistance is stable (Figs. 2, and S1 and S2) using linear stability analysis (see Methods). Under conditions selecting for resistance, we observe three behaviors: evolutionary stability of chromosomal—but not plasmid‐borne—resistance; evolutionary stability of plasmid‐borne—but not chromosomal—resistance; and evolutionary stability of both forms of resistance. In this third region, resistance occurs on either the plasmid or on the chromosome, but not both: having both chromosomal and plasmid‐borne resistance increases the cost of resistance while providing no added benefit.

#### Chromosomal resistance only

When only chromosomal resistance is evolutionarily stable, resistance genes will always end up on the chromosome over an evolutionary time‐scale. The plasmid will either be sensitive, or absent from the population. In general terms (Table 1 and Figs. S1 and   S2), chromosomal resistance is the only evolutionarily stable outcome when the benefit from resistance is high (high antibiotic associated mortality, low cost of resistance); when the fitness of the plasmid is low (low plasmid transmission rate, high segregation loss, high plasmid cost) and when overall cell density is low (high death rate, low replication rate).

#### Plasmid‐borne resistance only

When plasmid‐borne resistance is evolutionary stable, resistance genes will always end up on the plasmid. Note that in this region, chromosomal resistance is not stable at all (Fig. S12): it represents a region in which resistance genes can only persist when horizontally transferred (van Dijk et al. 2020). This outcome arises only under very specific conditions (small parameter space, when resistance yields only a minor fitness benefit; Fig. 2) and its presence is sensitive to model structure (e.g., how antibiotic effect is modeled; see Fig. S7). We therefore do not consider this an ecologically plausible explanation for why resistance genes are on plasmids.

#### Bistability

When both equilibria are evolutionarily stable, resistance can be either chromosomal or plasmid‐borne depending on initial conditions. Once one form of resistance has established, it can no longer be displaced by the other.

**Figure 3:**
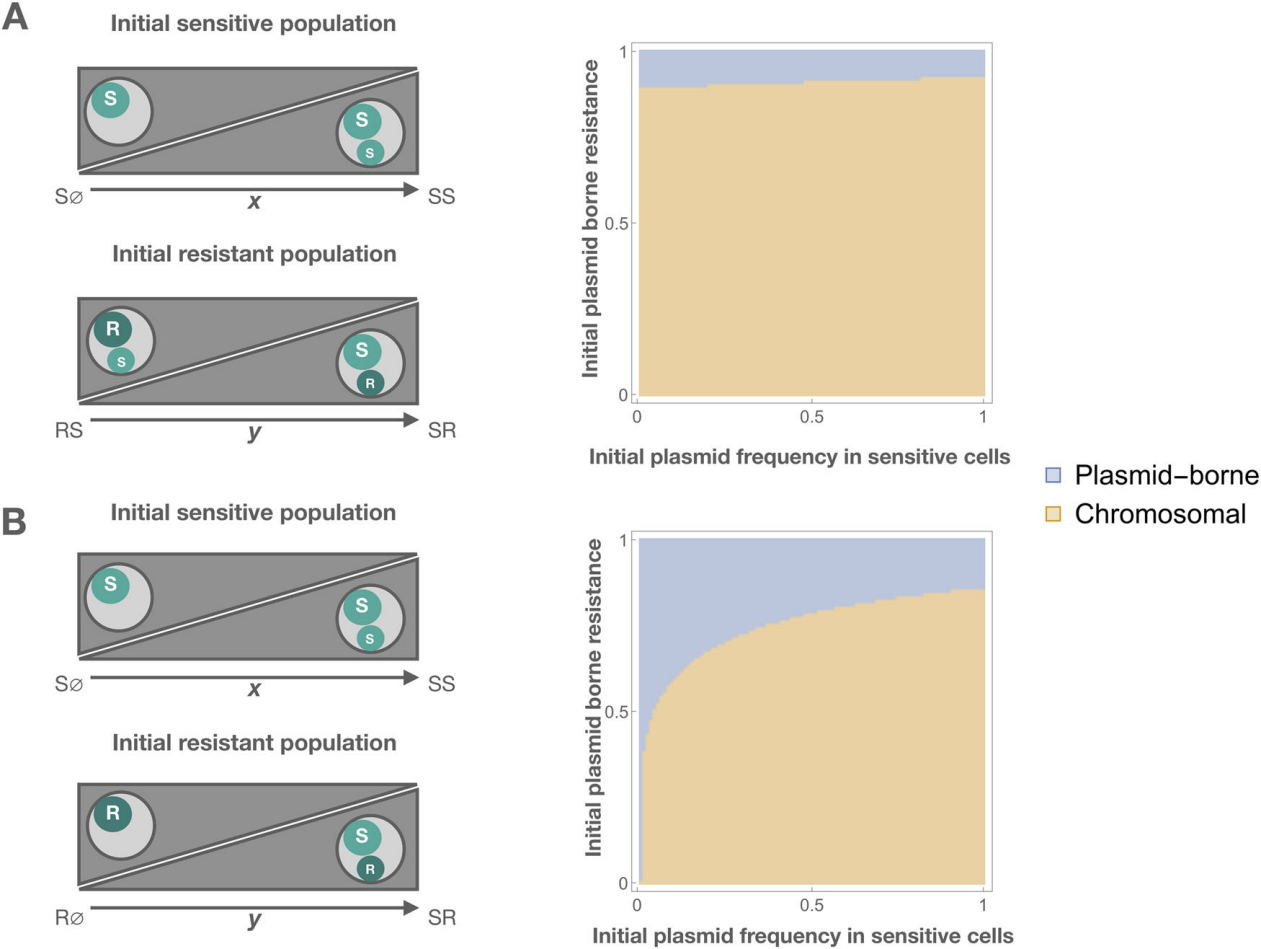
The effect of initial conditions on the equilibrium location of the resistance gene, showing evolutionary outcome depends on both the initial frequencies of plasmid‐borne and chromosomal resistance and the initial frequency of the sensitive plasmid. The left‐hand panels illustrate variation in the initial conditions. The right‐hand panels illustrate whether plasmid‐borne (blue) or chromosomal resistance (orange) is observed at equilibrium. The *x*‐axis indicates the frequency of the sensitive plasmid in the initial sensitive population $N_{SS}/\left(N_{S\emptyset }+N_{SS}\right)$. The *y*‐axis indicates the frequency of the plasmid‐borne resistance in the initial resistant population $N_{SR}/\left(N_{SR}+N_{RS}\right)$ for panel A, $N_{SR}/\left(N_{SR}+N_{R\emptyset }\right)$ for panel B. Plasmid‐borne resistance is a more typical outcome in panels B than A because of the presence of the sensitive plasmid in the initial chromosomally resistant population in panel A. The total densities of the initial sensitive and resistant populations are both 1. (Varying the initial ratio of resistance to sensitivity does not affect qualitative results—Fig. S3). Parameter values are as follows: λ=1, γ=1, s=0.005, $c_{P}$ = 0.075, β=0.2, A=1, and $c_{R}=0.05$.

To further investigate the dependence on initial conditions, we simulate the system numerically, starting at different initial cell densities (see Methods). We consider a scenario with an initial population consisting of resistant cells and sensitive cells (Fig. 3). We vary (i) the initial frequency of the sensitive plasmid in the sensitive population; (ii) the initial frequency of chromosomal versus plasmid‐borne resistance in the resistant population; and (iii) whether the chromosomally resistant cells carry the sensitive plasmid. The results of these simulations (Figs. 3 and S3 and S4) provide insight into the evolutionary pressures that determine the location of resistance genes in three ways.

First, the presence of positive frequency‐dependent selection: plasmid‐borne resistance is a more typical outcome when the initial frequency of plasmid‐borne resistance is high compared to the frequency of chromosomal resistance. Similarly, a high initial frequency of chromosomal resistance, compared to plasmid‐borne resistance, leads to chromosomal resistance as the evolutionary outcome. The fitness of one type of resistance is therefore positively correlated with its frequency. This frequency dependence arises because dually resistant cells are less fit than cells with either form of single resistance: dual resistance incurs an additional fitness cost but provides no additional fitness benefit. The higher the frequency of chromosomal resistance is, the higher the probability that a resistant plasmid will infect a chromosomally resistant (rather than chromosomally sensitive) cell. This disadvantages the resistant plasmid. Similarly, the higher the frequency of the resistant plasmid is, the higher the probability that a chromosomally resistant cell will be infected with the resistant plasmid. This disadvantages the resistant chromosome. Thus, the more common the resistance form, the greater its fitness compared to the other form.

Second, the evolutionary outcome also depends on the frequency of the sensitive plasmid. Plasmid‐borne resistance benefits from the sensitive plasmid being rare: plasmid‐borne resistance is a more typical outcome when the initial chromosomally resistant population does not carry the sensitive plasmid and when the frequency of the plasmid in the sensitive population is low. This is because a low initial frequency of the sensitive plasmid means that plasmid‐borne resistance can spread both vertically (cell replication) and horizontally (plasmid transmission), allowing it to increase in frequency more rapidly than chromosomal resistance.

Third, overall, chromosomal resistance is a more typical outcome in these simulations than plasmid‐borne resistance. This is because plasmid‐borne resistance, unlike chromosomal resistance, is subject to segregation loss: it is not always inherited during cell replication. Indeed, increasing the probability of segregation loss favors chromosomal resistance (Fig. S4).

### ROBUSTNESS OF RESULTS

We test the robustness of these results to a number of assumptions about model structure (see Methods and Supporting Information Section 2). The general result is that the presence of bistability is robust, although the size of the region of bistability can change. The only crucial assumption for positive frequency dependence is that dual resistance is less beneficial than single resistance (Figs. S5 and S6). In other words, eliminating the additional cost from dual resistance, or increasing the benefit of dual resistance so much that it outweighs this additional cost, abolishes the region of bistability. Under these circumstances, dual resistance dominates (i.e., the population will consist of a resistant plasmid circulating in a chromosomally resistant population).

The results of two sensitivity analyses in particular are worth highlighting. First, our results are robust to inclusion of gene flow between the plasmid and chromosome (e.g., transposition of the resistance gene). Gene flow allows the otherwise excluded form of resistance to persist at low frequency (analogous to mutation‐selection balance), and increases the range of initial conditions leading to chromosomal resistance (Fig. S8). However, these effects only become substantial for unrealistically high transposition rates (Supporting Information Section 2.4) (Sousa et al. 2013). Second, the presence of bistability is robust to modeling fluctuating, instead of constant, antibiotic pressure. Depending on its period, fluctuation can favor plasmid‐borne resistance, increasing the size of the parameter space in which only plasmid‐borne resistance is evolutionarily stable (Fig. S10).

### RELATIONSHIP TO PREVIOUS MODELING RESULTS

**Figure 4:**
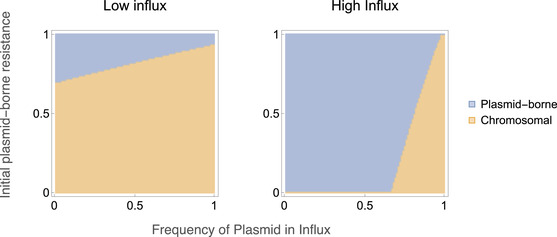
The location (chromosomal or plasmid‐borne) of a locally beneficial resistance gene depends on the presence of the sensitive plasmid in immigrant cells. The initial population is fully resistant (with chromosomally resistant cells carrying the sensitive plasmid, corresponding to panel A in Figure 3), with the *y*‐axis indicating the frequency of plasmid‐borne resistance in this initial population $N_{SR}/\left(N_{SR}+N_{RS}\right)$. The *x*‐axis indicates the frequency of the sensitive plasmid in the immigrant cells. The presence of the plasmid in these immigrant cells favors chromosomal resistance. The high influx rate is $\mu =10^{-1}$, the low influx rate is $\mu =10^{-2}$. Other parameters values are: λ=1, γ=1, s=0.005, $c_{P}$ = 0.075, β=0.2, A=1, and $c_{R}=0.05$.

Next, we revisit some previous modeling results. As discussed in the introduction, previous modeling predicts that locally beneficial traits will be plasmid‐borne rather than chromosomal, thus providing a complementary hypothesis for why certain genes reside on plasmids (Bergstrom et al. 2000). However, the model from which this prediction is derived assumes absence of the plasmid outside the local niche. We therefore ask whether local adaptation favors plasmid‐borne resistance if the sensitive plasmid can persist outside the local niche. We modify our model to include an influx of sensitive cells, and vary the frequency of the sensitive plasmid in these incoming cells (Supporting Information Section 3). This corresponds to a scenario in which resistance is locally beneficial in the modeled environment, but not selected for elsewhere. As shown in Figure 4, an influx of sensitive cells without the sensitive plasmid does indeed favor plasmid‐borne resistance, as suggested previously (Bergstrom et al. 2000). However, an influx of sensitive cells with the sensitive plasmid favors chromosomal resistance. The strength of this effect depends on the rate of influx of sensitive cells. Thus, local adaptation only favors plasmid‐borne resistance if the frequency of the sensitive plasmid is low outside the local niche.

Second, we revisit results relating to plasmid persistence. Previous modeling work has suggested that if plasmid fitness is too low for plasmids to persist as pure parasites (i.e., without carrying genes beneficial to the host cell), beneficial genes will always locate on the chromosome rather than plasmid (in absence of local adaptation; Bergstrom et al. 2000). Thus, the persistence of low transmissibility plasmids is a paradox: they cannot be maintained without beneficial genes, but beneficial genes cannot be maintained on these plasmids (Bergstrom et al. 2000).

We test this prediction in our model (as detailed in Supporting Information Section 4) by comparing the parameter space in which plasmid‐borne resistance is evolutionarily stable (i.e., resistance genes can locate onto the plasmid even in the presence of competition from chromosomal resistance) with the parameter space in which a parasitic plasmid can persist (i.e., a sensitive plasmid can persist in a chromosomally sensitive population). We find that previous results do not hold for the model structure presented here: resistance genes can locate onto the plasmid instead of the chromosome even if the plasmid transmissibility is too low for the plasmid to persist as a parasite (Fig. S11). This implies that in theory, it is possible for there to be low transmissibility plasmids which persist purely because of the advantage they provide host cells. It is worth noting, however, that the parameter space in which this occurs is small (Fig. S11).

### RATE OF ACQUISITION DETERMINES RESISTANCE GENE LOCATION

Thus far, our results show that for moderately beneficial genes (i.e., those in the bistable parameter region), the presence of positive frequency‐dependent selection means that plasmid‐borne resistance can be evolutionarily stable despite segregation loss. This frequency‐dependent selection is not, in itself, a sufficient explanation for why resistance genes are plasmid‐borne. However, it does suggest that whichever form of resistance (plasmid‐borne or chromosomal) is acquired first is likely to establish in the population: if the first form of resistance has time to increase in density prior to the acquisition of the other form, its greater frequency will give it a fitness advantage. The first resistance type need not have reached fixation to preclude invasion by the other: the frequency‐dependent advantage is sufficiently strong even at low overall resistance frequencies (Fig. S13). Therefore, when the rate of resistance acquisition is low compared to the rate of increase in resistance frequency once acquired, the first form of resistance will persist.

Thus, the presence of resistance genes on plasmids could be explained by the acquisition rate of plasmid‐borne resistance being higher than the acquisition rate of chromosomal resistance. Indeed, rates of conjugative plasmid transfer are generally higher than rates of chromosomal horizontal gene transfer (one estimate, based on comparison of experimental measures, suggests of the order of $10^{7}$ higher, though this is probably highly context dependent; Nazarian et al. 2018). Furthermore, for a number of bacterial species, the primary mechanism of resistance gene acquisition is indeed thought to be inter‐species transfer of resistance‐bearing plasmids (Baker et al. 2018; MacLean and San Millan 2019).

**Figure 5:**
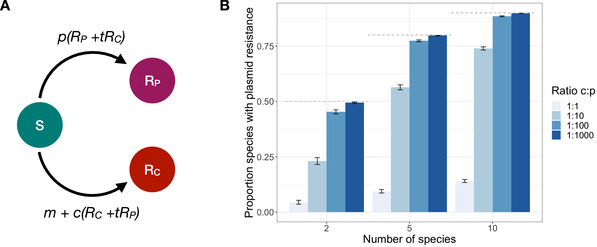
Prevalence of plasmid‐borne resistance. Panel A: Representation of the model structure. S represents species without the resistance gene; $R_{P}$ species with the resistance gene on the plasmid; $R_{C}$ species with the resistance gene on the chromosome. m is the rate at which resistance arises through mutation; p the rate at which the plasmid‐borne resistance is transferred between species; c the rate at which chromosomal resistance is transferred between species; and t captures gene flow between the plasmid and chromosome. Panel B: The proportion of plasmid‐borne resistance depends on the number of simulated species and the ratio of the rate of interspecies transfer of the chromosomal (c) and plasmid‐borne gene (p). The horizontal dashed lines show the maximum proportion of plasmid resistance, given that resistance must first emerge on the chromosome ((n−1)/n). The error bars represent 95% confidence intervals, based on 1000 realizations. Parameters were: $m=10^{-6}$, $c=10^{-5}$ and $t=10^{-1}$. Results for alternative parameterizations are shown in Figure S14.

To formalize this idea, we develop a simple model of resistance acquisition in multiple species (Fig. 5 and “Methods” section). We model n species; a resistance gene which is beneficial in all species; and a plasmid that can be transferred between and persist in all species (either because it has a broad host range or because its range can be shifted or expanded following transfer; Loftie‐Eaton et al. 2016). Resistance can be either plasmid‐borne or chromosomal. Once a species acquires one form of resistance, this form of resistance becomes established and can no longer be replaced (due to positive frequency‐dependent selection). We assume that resistance genes only emerge de novo on the chromosome (at rate m). The gene can spread through interspecies horizontal transfer of chromosomal resistance (e.g., transformation) (at rate c), or interspecies transfer of resistance plasmids (at rate p). We assume the gene can move between the plasmid and chromosome at low rates, which allows the otherwise excluded form of resistance to persist at low frequency. We do not explicitly model this coexistence, but do model the horizontal transfer of the low‐frequency form (at rate t∗p for plasmid‐borne resistance and rate t∗c for chromosomal resistance, where t is the frequency of the low frequency form).

We simulate this system stochastically (see “Methods” section), starting from no species having the resistance gene. Figure 5 shows the proportion of species with plasmid‐borne resistance once the gene has spread to all species. As expected, the proportion of species with plasmid‐borne resistance increases with the rate of interspecies plasmid transfer. In addition, this proportion also increases with the number of modeled species. This effect arises for two reasons. First, the initial de novo appearance of the gene must be on the chromosome. Thus, for example, when only two species are modeled, plasmid‐borne resistance can only occur in one of two species. Second, the impact of the rate of interspecies transfer increases with the number of potential donor species. These results are robust to different parametrization (Fig. S14).

## Discussion

To understand why certain gene functions, including antibiotic resistance, are found on plasmids, we develop a model of the competition between resistant and sensitive plasmids and resistant and sensitive chromosomes. Our key finding is that this model gives rise to positive frequency‐dependent selection on gene location. This positive frequency dependence arises when carrying both chromosomal and plasmid‐borne resistance is disadvantageous (i.e., the cost of the second copy outweights any additional reduction in antibiotic‐associated death). This disadvantage acts as a barrier to a low‐frequency form of resistance invading the population. Although the model was formulated in terms of the antibiotic resistance, we expect this central result to generalize to any gene where cells with both chromosomal and plasmid‐borne versions are at a disadvantage compared to cells with a single version.

The consequence of this frequency dependence is that for some parameter ranges genes can be maintained on plasmids, despite segregation loss, if they start with a frequency advantage. The key conditions are that the gene is only moderately beneficial—highly beneficial genes, whether strictly “essential”, are always chromosomal, because segregation loss is too detrimental—and that the fitness of the plasmid is not too low. Under these conditions, gene location (i.e., chromosomal or plasmid‐borne) depends on which form is acquired first. Using a simple stochastic model of resistance acquisition and transfer across multiple species, we show that the probability of finding genes on plasmids increases with the rate of interspecies plasmid transfer and with the number of species between which the gene and plasmid can be shared.

We also revisit the previously proposed idea that plasmids code for locally beneficial genes, and show that local adaptation does not explain plasmid‐borne resistance when the sensitive plasmid is present outside the local niche. Yet, many plasmid‐borne traits, such as antibiotic resistance, but also heavy metal tolerance (Piotrowska‐Seget et al. 2005) or metabolism of rare substances (Johnson and Nolan 2019), seem to fit the description of local or intermittent usefulness. Our results raise the possibility that these genes are on plasmids not because they code for local adaptation as such, but because their local usefulness means they are, on average, only moderately beneficial.

All together, the hypothesis that emerges from these results is that plasmid‐borne genes are (i) moderately beneficial, possibly due to heterogeneous selection pressure; (ii) functional across a large number of species; and (iii) rarely acquired through chromosomal mutation; and that resistance genes are often found on plasmids because these genes commonly fulfill these criteria.

Our model considers a single antibiotic and resistance gene. Resistance plasmids often carry multiple genes encoding resistance to different antibiotics (Nikaido 2009). As higher plasmid fitness favors plasmid‐borne resistance in our model, we might expect resistance genes to accumulate on highly transmissible plasmids. Furthermore, as acquisition of one beneficial gene increases plasmid fitness, such acquisition might allow the plasmid to aggregate further genes. However, it should also be noted that there are a number of other evolutionary mechanisms, in particularly correlated selection pressure for resistance against different antibiotics, which contribute to the co‐occurrence of resistance genes (Lehtinen et al. 2019; Jacopin et al. 2020).

Throughout the work, we have made the assumption that the effect of the gene of interest (e.g., for antibiotic resistance genes, their cost and effectiveness against antibiotic‐associated mortality) is the same on the chromosome and the plasmid: when this assumption does not hold, location can be trivially explained by the fitness difference. This assumption refers specifically to the effects of the same gene on the plasmid and chromosome when the gene is first introduced. (Note that a meta‐analysis of chromosomally acquired and plasmid‐acquired antibiotic resistance found the former to be costlier on average (Vogwill and MacLean 2015), but the analysis did not compare the cost of the *same* gene on the plasmid and chromosome.) It is relevant to consider this equal effect assumption in the light of both plasmid copy number and compensatory evolution.

First, the equal effects assumption would not hold for high copy number plasmids if gene expression levels increase with copy number: increased expression may lead to both higher cost (higher metabolic burden) and higher effectiveness. However, the extent to which gene expression increases with plasmid copy number is unclear because it depends on how tightly gene expression is regulated, which varies between plasmid genes (San Millan and MacLean 2019). Indeed, while there are some examples of phenotypic resistance increasing with copy number (e.g., amoxicillin‐clavulanate resistance in *Escherichia coli*; Davies et al. 2020), evidence from *Klebsiella pneumoniae* suggests that whether this effect is observed depends on both the antibiotic‐gene combination and the genetic background (van Dorp et al. 2019).

Second, compensatory evolution may eventually lead to genes having a different cost on the plasmid and chromosome, for example, through regulation of gene expression (Harrison et al. 2015; San Millan and MacLean 2019). However, we would not expect compensatory effects to be present when the gene is first introduced into the species. Indeed, compensatory evolution might subsequently reinforce the priority effect we observe, by giving the first form of resistance more time to acquire compensatory mutations (Vogwill and MacLean 2015).

The prediction of bistability is experimentally testable: this requires demonstrating that there are conditions under which resistance genes can be either chromosomal or plasmid‐borne, and that neither form of resistance can invade a population in which the other form of resistance is established. More specifically, our model predicts bistability when plasmid transmissibility is high and the benefit of the gene low: for lower transmissibility plasmids and a higher benefit from resistance (e.g., higher antibiotic concentration), we would expect chromosomal resistance to always invade.

Although we are not aware of studies directly testing these effects, the model predictions are consistent with existing experimental findings. First, previous experimental work has demonstrated that whether chromosomal resistance invades a population with plasmid‐borne resistance depends on plasmid transmissibility, with low plasmid transmissibility allowing invasion (Hall et al. 2016; Kottara et al. 2018). This is consistent with our model's predictions. However, demonstrating the presence of bi‐tability would require also showing that plasmid‐borne resistance cannot invade chromosomal resistance even when plasmid transmissibility is high.

Second, in a study of compensatory evolution to alleviate the fitness cost of a plasmid carrying mercury resistance, Harrison et al. (2015) found that the resistance gene frequently transitioned to the chromosome. These chromosomally resistant lineages lost the plasmid, but were generally not able to increase in frequency. The authors attributed the effect to compensatory evolution alleviating the cost of the plasmid. However, unless the cost of the plasmid was fully alleviated, the chromosomally resistant and plasmid‐free lineage would still be fitter than the plasmid‐bearing lineage and thus expected to invade. Yet, if the cost of the plasmid was fully alleviated, the chromosomally resistant lineage would not necessarily lose the plasmid. Therefore, the inability of these chromosomally resistant cells to invade is compatible with both compensatory adaptation and frequency‐dependent selection.

Rodríguez‐Beltrán et al. (2020) have recently shown that polyploidy (i.e., gene copies being present on both mobile element and chromosome or, for multi‐copy plasmids, on multiple plasmids) plays an important, and thus far neglected, role in mobile genetic element evolution: polyploidy masks the effect of recessive mutations on mobile genetic elements (genetic dominance), and thus mobile genetic elements are primarily associated with dominant mutations. Our results can also be thought of in terms of polyploidy: positive frequency dependence arises because polyploidy has different effects on the cost and effectiveness of the gene. Our results are therefore another example of the emerging importance of polyploidy effects in understanding the gene content of plasmids.

Finally, our results provide a new perspective on the extent to which the long‐term fate of resistance genes depends on stochastic gene acquisition events. Recent analysis of resistance dynamics in *Streptococcus pneumoniae* has suggested that the rate at which lineages acquire resistance genes is not a major determinant of their resistance frequencies. This supports the view that resistance evolution is a deterministic outcome of selection pressures (Lehtinen et al. 2020). Our results provide a different perspective: in the context of the location of resistance genes, the priority effect arising from positive frequency‐dependent selection means that the timing of gene acquisition events sets the population onto an evolutionary path from which it cannot subsequently deviate. Thus, in this context, eventual evolutionary outcomes are fundamentally stochastic.

## Methods

### COMPUTATION OF EQUILIBRIA AND STABILITY ANALYSIS

We consider the dynamics of a population consisting of cells that can be either chromosomally resistant or chromosomally sensitive, with no plasmid, a resistant plasmid, or a sensitive plasmid, giving rise to a model with six cell types (eq. 1 and Fig. 1). Our aim is to study the evolutionary stability of plasmid‐borne and chromosomal resistance by finding the equilibria of this system and determining their stability. We were unable to solve for the equilibria of the full six species system. However, we can use insights into dynamics of the system to simplify the problem. First, we do not expect coexistence of the sensitive and resistant chromosome, nor of the sensitive and resistant plasmid: in each case, the sensitive and resistant variant are competing for the same resource and thus, without a coexistence promoting mechanism, we expect to see competitive exclusion (Chesson 2000; Colijn et al. 2010; Lehtinen et al. 2017). Second, we do not expect presence of cells with both plasmid‐borne and chromosomal resistance at equilibrium (RR): because dual resistance carries a dual cost, but not a dual benefit, these cells will always be inferior to cell with single resistance (R∅, RS, or SR).

This allows us to narrow the possible equilibria in which resistance is present to three: presence of chromosomal resistance, either in presence or absence of the sensitive plasmid ($N_{R\emptyset }>0,N_{RS}>0$, all other cell types 0; or $N_{R\emptyset }>0$, all other cell types 0), and plasmid‐borne resistance ($N_{S\emptyset }>0,N_{SR}>0$, all other cell types 0). For each equilibrium of interest, therefore, we reduce the full model by setting the relevant subset of the variables to zero and compute the steady state of this reduced system. We then check the stability of this steady state in the *full model* by computing the eigenvalues of the full model's Jacobian evaluated at this equilibrium point. The parameter region in which all six eigenvalues are negative is the region in which the equilibrium is evolutionarily stable.

These calculations were implemented in Wolfram Mathematica 12 (Inc WR 2017). The code is available as a Supporting File . Note that our reasoning for narrowing down the equilibrium of interest is verbal rather than a mathematical: we can therefore not rule out the possibility we have overlooked a relevant equilibrium. However, such equilibria are never seen in any of our numerical simulations.

### NUMERICAL SIMULATIONS OF DYNAMICS

For the analyses examining the dependence of resistance on initial conditions, we simulate the system numerically until equilibrium is reached (for $10^{7}$ timesteps unless otherwise indicated). To avoid nonzero cell densities arising from numerical errors, final states are rounded to $10^{-10}$. These simulations were implemented in Wolfram Mathematica 12 Inc WR (2017). The code is available as a Supporting File.

### SENSITIVITY ANALYSES

We perform sensitivity analyses to test the robustness of our results to a number of changes in model structure: modifying the effect of dual resistance (decreasing the cost, increasing the benefit); modeling segregation loss independently from replication; modeling the antibiotic as slowing growth rate rather than increasing death rate; modeling fitness costs as increased death rate rather than decreased growth rate; allowing gene flow between the plasmid and chromosome; and relaxing the assumption that carriage of one plasmid completely excludes the other. Full details are provided in Supporting Information Section 2.

### MULTISPECIES MODEL

For the multi‐species model of resistance acquisition, we model n species that can be in one of three states: without resistance (S), with chromosomal resistance ($R_{C}$) or with plasmid‐borne resistance ($R_{P}$). Once species have reached either state $R_{C}$ or $R_{P}$, they remain in this state due to frequency‐dependent selection favoring the first acquired form of resistance. Species can transition from state S to state $R_{C}$ through *de novo* mutation (at rate m) or transfer of the resistance gene from a species with chromosomal resistance (at rate $cR_{C}$). In addition, we allow for gene flow between the plasmid and chromosome leading to the otherwise excluded form of resistance persisting at low frequency. We do not explicitly model this coexistence, but do model the horizontal transfer of the low frequency form. Thus, species can also transition from state S to state $R_{C}$ through transfer from a species with plasmid‐borne resistance (at rate t∗c, where t captures the frequency of the low‐frequency form arising from gene movement between the plasmid and chromosome). Species can transition from state S to $R_{P}$ through interspecies plasmid transfer, either from a species with plasmid‐borne resistance (at rate p) or chromosomal resistance (at rate t∗p).

We simulate this model stochastically, starting from all species in state S, until all species have acquired resistance. If $N_{R_{C}}$ is the number of species with chromosomal resistance and $N_{R_{P}}$ is the number of species with plasmid‐borne resistance, then, at any given timestep, the probability of any given species transitioning from the S state to one of the R states is given by

(2) $$\begin{array}{ccc} P(S\to R_{C}) & = & m+cN_{R_{C}}+ctN_{R_{P}}\\ P(S\to R_{P}) & = & pN_{R_{P}}+ptN_{R_{C}}. \end{array}$$

Figure 5 is based on 1000 realizations. The simulation was implemented in R The R Development Core Team (2013) and the code is available as a Supporting File.

## 1 AUTHOR CONTRIBUTIONS

All authors jointly conceived the study. S.L. performed the modelling and analysis and wrote the first draft of the manuscript. All authors reviewed and edited the final manuscript.

## DATA ARCHIVING

The data underlying this article are available in the article and in its online Supporting Information.

Associate Editor: K. Lythgoe

## Supporting information


**Figure 1**: Evolutionary stability of plasmid‐borne and chromosomal resistance.
**Figure 2**: Evolutionary stability of plasmid‐borne and chromosomal resistance at lower antibiotic consumption rate (A=0.5), where sensitive cells are viable the standard value of the replication rate (λ=1).
**Figure 3**: Effect of initial conditions on equilibrium location of resistance gene, with initially low resistance frequency.
**Figure 4**: Effect of parametrization on the relationship between initial conditions and evolutionary outcomes.
**Table 1**: Summary of sensitivity analyses and their effects on model behaviour.
**Figure 5**: Effect of decreasing the cost incurred from a second copy of the resistance gene.
**Figure 6**: Effect of assuming a single copy of the resistance does not fully prevent antibiotic‐ associated mortality.
**Figure 7**: Robustness of model results to assumptions about segregation loss, the effect of antibiotic action and how fitness costs are modeled.
**Figure 8**: Effect of gene ow between plasmid and chromosome.
**Figure 9**: Effect of initial conditions on equilibrium location of the resistance gene, with imperfect exclusion of plasmid co‐infection.
**Figure 10**: The effect of uctuating selection on the evolutionary stability of chromosomal and plasmid‐borne resistance: the presence of bistability is robust, with plasmid‐borne resistance favoured under some conditions.
**Figure 11**: Resistance can locate onto the plasmid even when plasmid transmissibility is low, allowing low transmissibility plasmids to persist because of the advantage they provide their host cells.
**Figure 12**: Stability of plasmid‐borne and chromosomal resistance in absence of competition from the other resistance form (but in presence of the sensitive chromosome and sensitive plasmid).
**Figure 13**: Low densities of plasmid‐borne resistance are enough to prevent invasion by chromosomal resistance.
**Figure 14**: Spread of resistance genes between species for rate parameters differing from those used to generate Figure 5 in the main text.

Supplementary Material
