# Supplementary material for: Evolutionary mechanisms that determine which bacterial genes are carried on plasmids
Source: Evol Lett. 2021 May 18;5(3):290–301. doi: 10.1002/evl3.226 (PMC8190454; doi:10.1002/evl3.226)
Supplement: Supplementary file 1 — Figure 1: Evolutionary stability of plasmid‐borne and chromosomal resistance. Figure 2: Evolutionary stability of plasmid‐borne and chromosomal resistance at lower antibiotic consumption rate (A=0.5), where sensitive cells are viable the standard value of the replication rate (λ=1). Figure 3: Effect of initial conditions on equilibrium location of resistance gene, with initially low resistance frequency. Figure 4: Effect of parametrization on the relationship between initial conditions and evolutionary outcomes. Table 1: Summary of sensitivity analyses and their effects on model behaviour. Figure 5: Effect of decreasing the cost incurred from a second copy of the resistance gene. Figure 6: Effect of assuming a single copy of the resistance does not fully prevent antibiotic‐ associated mortality. Figure 7: Robustness of model results to assumptions about segregation loss, the effect of antibiotic action and how fitness costs are modeled. Figure 8: Effect of gene ow between plasmid and chromosome. Figure 9: Effect of initial conditions on equilibrium location of the resistance gene, with imperfect exclusion of plasmid co‐infection. Figure 10: The effect of uctuating selection on the evolutionary stability of chromosomal and plasmid‐borne resistance: the presence of bistability is robust, with plasmid‐borne resistance favoured under some conditions. Figure 11: Resistance can locate onto the plasmid even when plasmid transmissibility is low, allowing low transmissibility plasmids to persist because of the advantage they provide their host cells. Figure 12: Stability of plasmid‐borne and chromosomal resistance in absence of competition from the other resistance form (but in presence of the sensitive chromosome and sensitive plasmid). Figure 13: Low densities of plasmid‐borne resistance are enough to prevent invasion by chromosomal resistance. Figure 14: Spread of resistance genes between species for rate parameters differing from those used to generate Figure 5 in the ma [file EVL3-5-290-s001.pdf]

Supporting Information

1 Parametrisation

1.1 Evolutionary Stability

SI Figures 1 and 2 explore the evolutionary stability of plasmid-borne and chromosomal resistance for a wide range of parameter combinations. Each figure is associated with a set of standard parameter values, with each panel exploring variation around these standard values for one pair of parameters. For SI Figure 1, the standard parameter values are the same as in main text Figure 2: ($\lambda = 1$, $\gamma = 1$, $s = 0.005$, $c_P = 0.075$, $\beta = 0.2$, $A = 1$ and $c_R = 0.05$). SI Figure 2 explores behaviour at a lower antibiotic consumption rate ($A = 0.5$), where sensitive cells are viable at the standard value of the replication rate ($\lambda = 1$). The two figures are very similar, with minor differences for some panels in which the fitness cost of resistance is varied. Thus, the distinction between essential and simply beneficial resistance is not meaningful in our model.

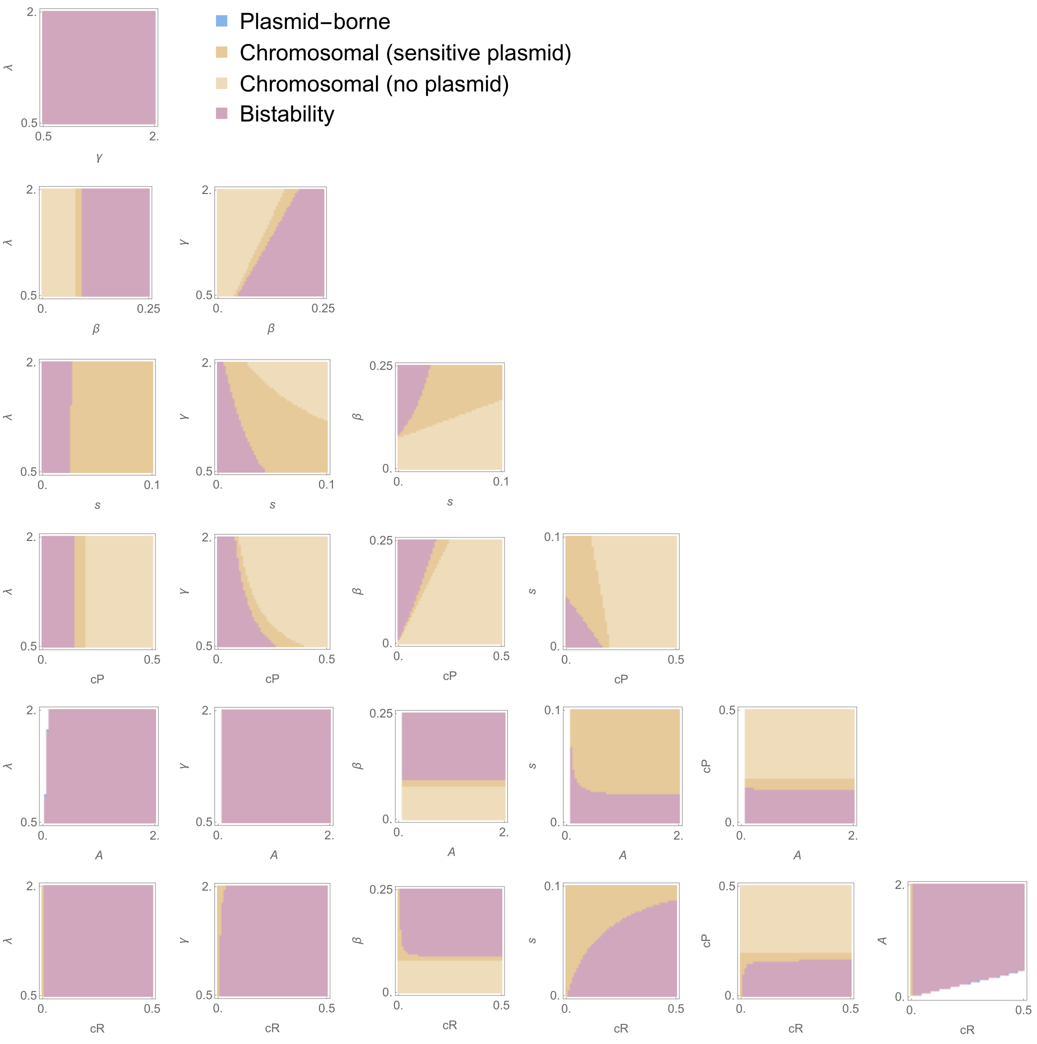

Figure 1: Evolutionary stability of plasmid-borne and chromosomal resistance. Each panel shows parameter regions in which plasmid-borne only (blue); chromosomal resistance (orange); or both (purple) are evolutionarily stable, for varying values of a pair of parameters. For each panel, values for non-varying parameters are the same as in main text: $\lambda = 1$, $\gamma = 1$, $s = 0.005$, $c_P = 0.075$, $\beta = 0.2$, $A = 1$ and $c_R = 0.05$.

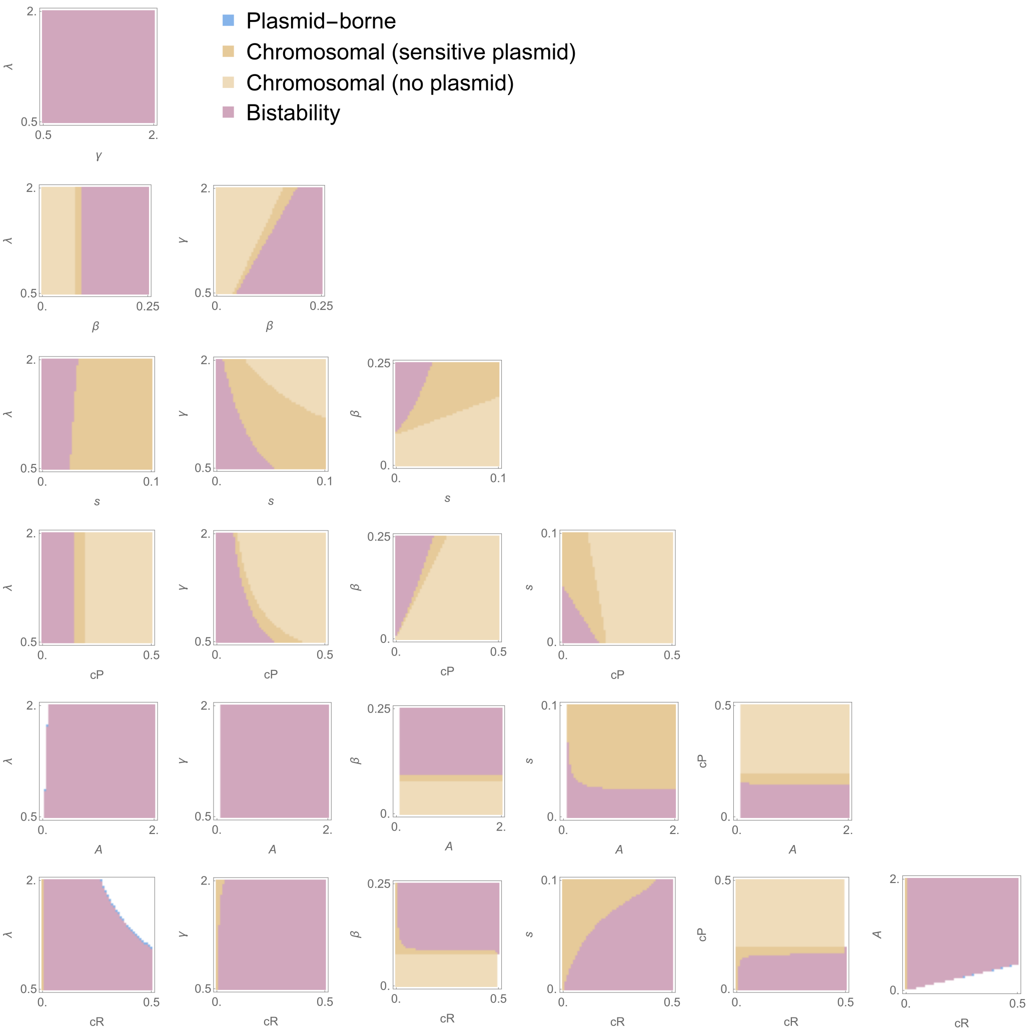

Figure 2: Evolutionary stability of plasmid-borne and chromosomal resistance at lower antibiotic consumption rate ($A = 0.5$), where sensitive cells are viable the standard value of the replication rate ($\lambda = 1$). Each panel shows parameter regions in which plasmid-borne only (blue); chromosomal resistance (orange); or both (purple) are evolutionarily stable, for varying values of a pair of parameters. For each panel, values for non-varying parameters are the same as in main text, except that antibiotic consumption is lower (and cells without the resistance gene are therefore viable): $\lambda = 1$, $\gamma = 1$, $s = 0.005$, $c_P = 0.075$, $\beta = 0.2$, $A = 0.5$ and $c_R = 0.05$.

1.2 Dependence on initial conditions

SI Figures 3 and 4 explore how the relationship between initial conditions and evolutionary outcomes depends on parametrisation. The default parameters are the same as in the main text ($\lambda = 1$, $\gamma = 1$, $s = 0.005$, $c_P = 0.075$, $\beta = 0.2$, $A = 1$ and $c_R = 0.05$), with variation indicated in figure labels and legends. The relationship between parameter values and evolutionary outcome is the same as found in the analysis of evolutionary stability, i.e. plasmid-borne resistance is favoured by:

- high replication rate (λ), plasmid-transmission rate (β), and high cost of resistance (c_R)
- low density-dependent death rate (γ), segregation loss (s), cost of plasmid (c_P), and antibiotic associated mortality (A).

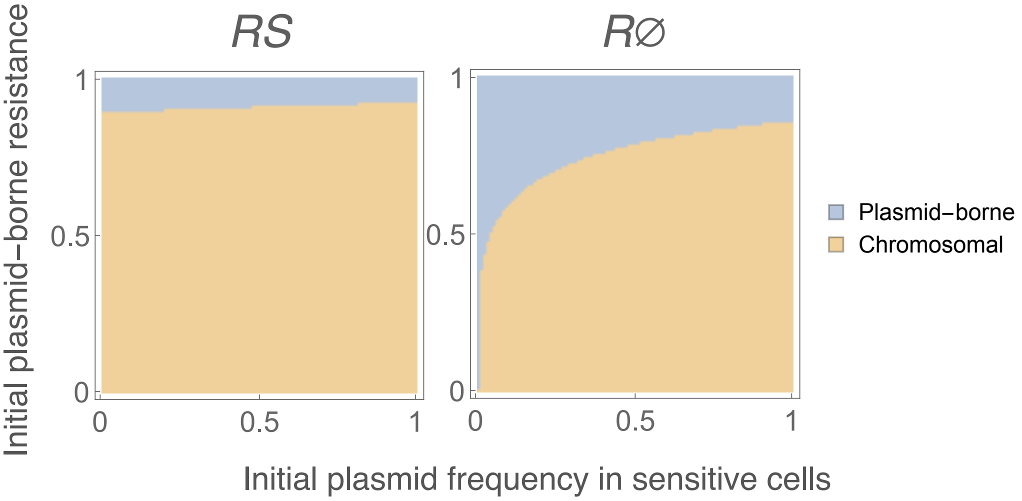

Figure 3: Effect of initial conditions on equilibrium location of resistance gene, with initially low resistance frequency. In main text Figure 3, the initial densities of the sensitive and resistant population are both 1. Here, the initial density of the sensitive population is 1, and the initial density of the resistant population is 0.01. As in main text Figure 3, the panels illustrates whether plasmid-borne (blue) or chromosomal resistance (orange) are observed at equilibrium. The x-axis indicates the frequency of the sensitive plasmid in the initial sensitive population. The y-axis indicates the frequency of the plasmid-borne resistance in the initial resistant population. For the left hand panel, the initial chromosomally resistant population carries the sensitive plasmid (RS), for the right hand panel, it does not ($R\emptyset$). Parameter values are: $\lambda = 1$, $\gamma = 1$, $s = 0.005$, $c_P = 0.075$, $\beta = 0.2$, $A = 1$ and $c_R = 0.05$.

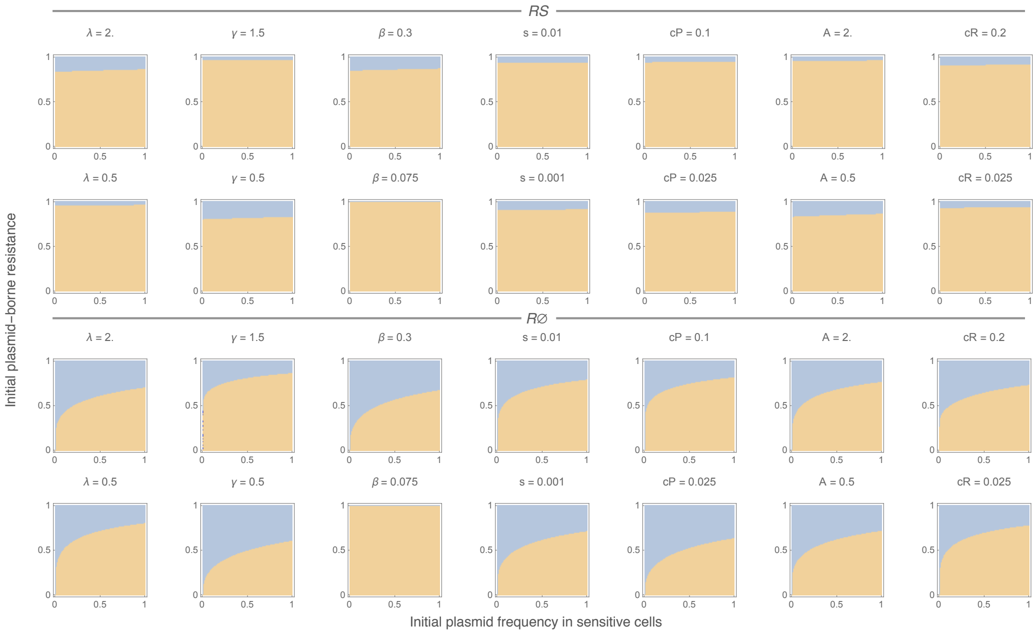

Figure 4: Effect of parametrisation on the relationship between initial conditions and evolutionary outcomes. Each panel depicts the effect of changing one parameter from the default parametrisation ($\lambda = 1$, $\gamma = 1$, $s = 0.005$, $c_P = 0.075$, $\beta = 0.2$, $A = 1$ and $c_R = 0.05$). As in main text Figure 3, the panels illustrates whether plasmid-borne (blue) or chromosomal resistance (orange) are observed at equilibrium. The x-axis indicates the frequency of the sensitive plasmid in the initial sensitive population. The y-axis indicates the frequency of the plasmid-borne resistance in the initial resistant population. The total densities of the initial sensitive and resistant populations are both 1. Top rows: chromosomally resistant cells in the initial population carry the sensitive plasmid. Bottom rows: chromosomally resistant cells in the initial population do not carry the sensitive plasmid. For a small number of initial conditions, the numerical simulation of the system failed due to problems with precision ($\gamma = 1.5$ with initial $R\emptyset$ population, panel in second column, third row). These simulations are indicated in dark blue.

2 Sensitivity Analyses

In this section, we test the robustness of our results to assumptions about model structure. SI Table 1 summarises the analyses and their effect on our key results.

**Sensitivity analysis**	**Positive frequency-dependent selection (i.e. region of bi-stability)**	**Other effects**
Decreasing cost of dual resistance	Not observed when dual resistance incurs no additional cost	Favours chromosomal resistance
Increasing benefit of dual resistance	Not observed when benefit of dual resistance out-weights its cost	Favours chromosomal resistance
Independent segregation loss	Robust	Favours chromosomal resistance
Antibiotic effect on growth	Robust	Removes parameter space in which plasmid-borne resistance alone is stable
Fitness costs as death rate	Robust	Favours chromosomal resistance; removes parameter space in which plasmid-borne resistance alone is stable
Gene flow between plasmid and chromosome	Robust	Allows low level coexistence of both resistance forms; favours chromosomal resistance at high levels of gene flow
Imperfect exclusion of plasmid co-infection	Robust	Favours plasmid-borne resistance
Fluctuating antibiotic selection	Robust	Favours plasmid-borne resistance under some conditions

Table 1: Summary of sensitivity analyses and their effects on model behaviour.

Note that the parametrisation for the figures in this Section (SI Figures 5 to 7) is slightly different from that used the main text: the plasmid transmission rate in the main text is $\beta = 0.2$, whereas here we use $\beta = 0.10$. This is to more clearly illustrate that the parameter region at low cost of resistance in which only chromosomal resistance is evolutionarily stable also arises in the main model structure. In other words, that this effect is not a consequence of modifying the model structure. This region is also present when $\beta = 0.2$ (Main Text Figure 2), but is very small and therefore difficult to see.

2.1 Cost of second resistance gene

SI Figure 5 shows the effect of decreasing the cost incurred from the second copy of the resistance gene. The model structure remains as described by Equations 1 in the main text. However, the fitness cost of the second resistance gene is modulated by a factor e. Thus, the replication rate of dually resistant cells is given by: $\lambda_{RRP} = (1 - e * c_R)(1 - c_R)(1 - c_P)\lambda$. Setting $e = 1$ recovers the model from the main text, whereas $e = 0$ means a second copy of the resistance incurs no additional fitness cost. Note that if $e = 0$, resistance will occur on both the chromosome and plasmid.

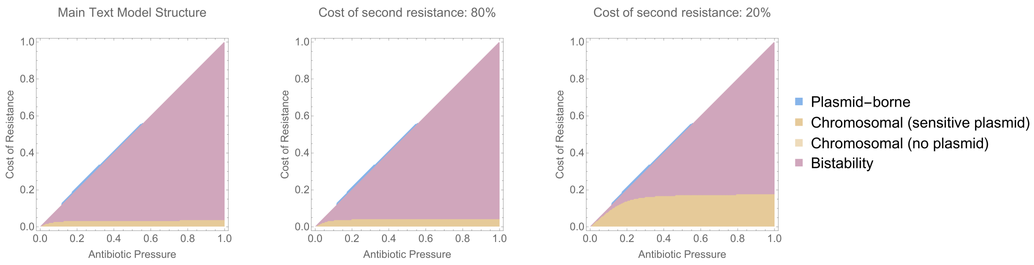

Figure 5: Effect of decreasing the cost incurred from a second copy of the resistance gene. Each panel shows parameter regions in which plasmid-borne only (blue); chromosomal resistance (orange); or both (purple) are evolutionarily stable, as a function of antibiotic consumption and the cost of resistance. Left-hand panel: $e = 1$ (main text model); middle panel: $e = 0.8$; right-hand panel: $e = 0.2$. Other parameters are: $\lambda = 1$, $\gamma = 1$, $s = 0.005$, $c_P = 0.075$, and $\beta = 0.1$. Note that the value of β is different from the value in the main text ($\beta = 0.2$) to better illustrate the region of chromosomal resistance only at low cost of resistance. With $e = 0$, resistance occurs on both chromosome and plasmid.

2.2 Benefit of second resistance gene

SI Figure 6 shows the effect of assuming a single copy of the resistance does not fully prevent antibiotic-associated mortality. Cells with a single copy of the resistance gene (i.e. either chromosomal or plasmid-borne resistance only) are subject to an antibiotic-associated mortality of $(1-a)A$, where a parametrises the effectiveness of a single resistance gene. Setting $a = 1$ recovers the model from the main text, whereas $a = 0$ would mean a single copy of the gene does not decrease antibiotic associated mortality at all. The dynamics of this modified model (with changes from the main text highlighted in bold) are described by:

$$\begin{aligned}
\frac{dN_{S\emptyset}}{dt} &= N_{S\emptyset}[\lambda - \beta(N_{SS} + N_{SR} + N_{RS} + N_{RR}) - \gamma T - A] + s(\lambda_P N_{SS} + \lambda_{RP} N_{SR}) \\
\frac{dN_{SS}}{dt} &= N_{SS}[(1-s)\lambda_P - \gamma T - A] + \beta(N_{SS} + N_{RS})N_{S\emptyset} \\
\frac{dN_{SR}}{dt} &= N_{SR}[(1-s)\lambda_{RP} - \gamma T - \mathbf{(1\text{-}a)A}] + \beta(N_{SR} + N_{RR})N_{S\emptyset} \\
\frac{dN_{R\emptyset}}{dt} &= N_{R\emptyset}[\lambda_R - \beta(N_{SS} + N_{SR} + N_{RS} + N_{RR}) - \gamma T - \mathbf{(1\text{-}a)A}] + s(\lambda_{RP} N_{RS} + RRN_{RR}) \\
\frac{dN_{RS}}{dt} &= N_{RS}[(1-s)\lambda_{RP} - \gamma T - \mathbf{(1\text{-}a)A}] + \beta(N_{SS} + N_{RS})N_{R\emptyset} \\
\frac{dN_{RR}}{dt} &= N_{RR}[(1-s)\lambda_{RRP} - \gamma T] + \beta(N_{SR} + N_{RR})N_{R\emptyset}
\end{aligned} \tag{1}$$

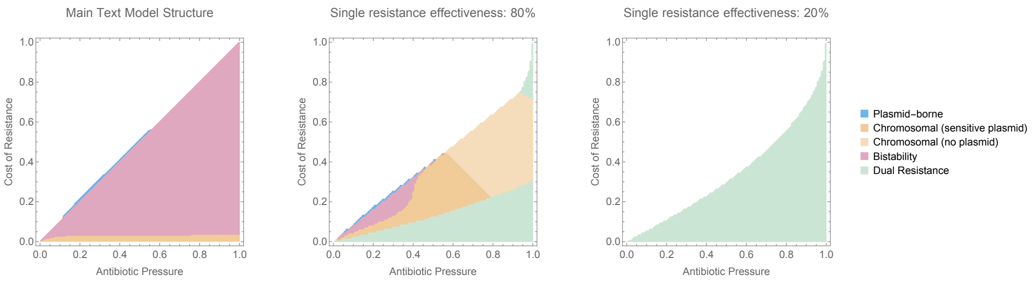

Figure 6: Effect of assuming a single copy of the resistance does not fully prevent antibiotic-associated mortality. Each panel shows parameter regions in which plasmid-borne only (blue); chromosomal resistance (orange); or both (purple) are evolutionarily stable, as a function of antibiotic consumption and the cost of resistance. Left-hand panel: $a = 1$ (main text model); middle panel: $a = 0.8$; right-hand panel: $a = 0.2$. Other parameters are: $\lambda = 1$, $\gamma = 1$, $s = 0.005$, $c_P = 0.075$, and $\beta = 0.1$. Note that the value of β is different from the value in the main text ($\beta = 0.2$) to better illustrate the region of chromosomal resistance only at low cost of resistance.

2.3 Other features of model structure

SI Figure 7 explores the robustness of our results to other aspects of model structure. Although the predicted outcome at specific parameter values depends on model structure, the key result, that there is a parameter region of bi-stability where either plasmid-borne or chromosomal resistance can occur, is robust.

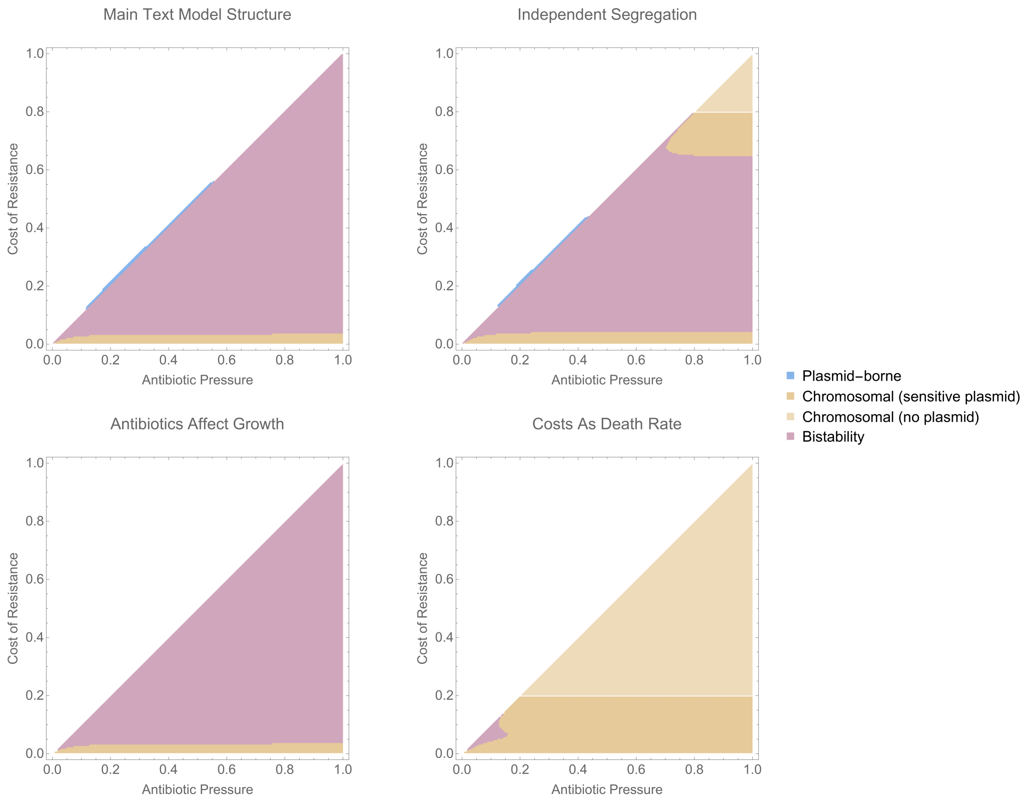

Figure 7: Robustness of model results to assumptions about segregation loss, the effect of antibiotic action and how fitness costs are modelled. Although the predicted outcome at specific parameter values depends on model structure, the key result that there is an area of bi-stability where either plasmid-borne or chromosomal resistance can occur is robust. Parameters are: $\lambda = 1$, $\gamma = 1$, $s = 0.005$, $c_P = 0.075$, and $\beta = 0.1$. Note that the value of β is different from the value in the main text ($\beta = 0.2$) to better illustrate the region of chromosomal resistance only at low cost of resistance.

2.3.1 Independent segregation loss

In the main text, segregation loss is modelled as occurring during cell replication. Some previous models addressing similar topics (Svara & Rankin [main text reference 6] and Bergstrom et al. [main text ref 16]) have modelled segregation loss as occurring independently of cell replication. We therefore verify that our results are robust to modelling segregation loss in this manner. The dynamics of the modified model (with the difference from the main text highlighted in bold) are given by:

$$\begin{aligned}
\frac{dN_{S\emptyset}}{dt} &= N_{S\emptyset}[\lambda - \beta(N_{SS} + N_{SR} + N_{RS} + N_{RR}) - \gamma T - A] + \mathbf{s}(\mathbf{N_{SS}} + \mathbf{N_{SR}}) \\
\frac{dN_{SS}}{dt} &= N_{SS}[\lambda_P - \gamma T - A - \mathbf{s}] + \beta(N_{SS} + N_{RS})N_{S\emptyset} \\
\frac{dN_{SR}}{dt} &= N_{SR}[\lambda_{RP} - \gamma T - \mathbf{s}] + \beta(N_{SR} + N_{RR})N_{S\emptyset} \\
\frac{dN_{R\emptyset}}{dt} &= N_{R\emptyset}[\lambda_R - \beta(N_{SS} + N_{SR} + N_{RS} + N_{RR}) - \gamma T] + \mathbf{s}(\mathbf{N_{RS}} + \mathbf{N_{RR}}) \\
\frac{dN_{RS}}{dt} &= N_{RS}[\lambda_{RP} - \gamma T - \mathbf{s}] + \beta(N_{SS} + N_{RS})N_{R\emptyset} \\
\frac{dN_{RR}}{dt} &= N_{RR}[\lambda_{RRP} - \gamma T - \mathbf{s}] + \beta(N_{SR} + N_{RR})N_{R\emptyset}
\end{aligned} \tag{2}$$

Our key result, the presence of bi-stability, is robust to this change in model structure. However, the relationship between the cost of resistance and evolutionary outcome is altered. In the main text model structure, bi-stability occurs once the cost of resistance is high enough. In this modified model, chromosomal stability is the only evolutionarily stable outcome at low *and high* cost of resistance, with bi-stability in between.

2.3.2 Antibiotic effect in growth

In the main text, we model antibiotic action as an additional mortality rate (*bactericidal*). Antibiotics can also have the effect stopping cell replication (*bacteriostatic*). To test the robustness of our results to mechanism of antibiotic action, we modify the effect of antibiotics so that they decrease growth rate. The model structure remains otherwise similar to Equations 1 in the main text, but the growth rate of antibiotic sensitive cells is decreased by a factor of $1 - A$. Thus the growth rate of S cells, previously λ, becomes $(1 - A)\lambda$, and the growth rate of SS cells, previously λ_P, becomes $(1 - A)\lambda_P$.

This change eliminates the region in which plasmid-borne resistance is stable but chromosomal resistance is not, but does not otherwise impact our results. Thus, our main findings apply to both bacteriostatic and bactericidal antibiotics. More generally, this broadens the relevance of our results from antibiotic resistance genes to any gene with effects on either the growth or death rate of a cell.

2.3.3 Additive costs

In the main text, we model the fitness cost of resistance as a multiplicative factor on the growth rate. Here, we modify the model so that the cost of antibiotic resistance and plasmid carriage is modelled as an additional death rate. They dynamics of this modified system are described by the following equations (differences from the main text are highlighted):

$$\begin{aligned}
\frac{dN_{S\emptyset}}{dt} &= N_{S\emptyset}[\lambda - \beta(N_{SS} + N_{SR} + N_{RS} + N_{RR}) - \gamma T] + s(\lambda N_{SS} + \lambda N_{SR}) \\
\frac{dN_{SS}}{dt} &= N_{SS}[(1-s)\lambda - \mathbf{c_P} - \gamma T] + \beta(N_{SS} + N_{RS})N_{S\emptyset} \\
\frac{dN_{SR}}{dt} &= N_{SR}[(1-s)\lambda - \mathbf{c_P} - \mathbf{c_R} - \gamma T] + \beta(N_{SR} + N_{RR})N_{S\emptyset} \\
\frac{dN_{R\emptyset}}{dt} &= N_{R\emptyset}[\lambda - \mathbf{c_R} - \beta(N_{SS} + N_{SR} + N_{RS} + N_{RR}) - \gamma T] + s(\lambda N_{RS} + \lambda N_{RR}) \\
\frac{dN_{RS}}{dt} &= N_{RS}[(1-s)\lambda - \mathbf{c_P} - \mathbf{c_R} - \gamma T] + \beta(N_{SS} + N_{RS})N_{R\emptyset} \\
\frac{dN_{RR}}{dt} &= N_{RR}[(1-s)\lambda - \mathbf{c_P} - \mathbf{2c_R} - \gamma T] + \beta(N_{SR} + N_{RR})N_{R\emptyset}
\end{aligned} \tag{3}$$

This change also eliminates the region in which plasmid-borne resistance is stable but chromosomal resistance is not. The region of bi-stability is still observed, although for this parametrisation, the region is considerably smaller. However, it should be noted that although the parameter values are the same as in the main text model, the parametrisations are no longer directly comparable because the scaling of the fitness cost is no longer the same: in the main text, fitness cost is constrained to be between 0 and 1, whereas here, the value is constrained but its effective magnitude depends on the replication and death rates (i.e. a cost of $c_R = 0.5$ for example has different impact if $\lambda = 0.5$ than if $\lambda = 2$).

2.4 Gene flow between plasmid and chromosome

To model the effect of genes on transposons moving between the plasmid and chromosome, we modify the model (main text Equations 1) to include a transposition term. We model replicative ('copy-paste') transposition: SR cells convert to RR cells at rate $\rho\lambda_{PR}$, and RS cells convert to RR cells at rate $\rho\lambda_{PR}$. The transposition rate ρ is scaled with replication rate to allow easy comparison of modelled transposition rates to empirical estimates expressed in terms of transposition events per generations (see below). The dynamics of the modified system are captured by:

$$
\begin{aligned}
\frac{dN_{S\emptyset}}{dt} &= N_{S\emptyset}[\lambda - \beta(N_{SS} + N_{SR} + N_{RS} + N_{RR}) - \gamma T - A] + s(\lambda_P N_{SS} + \lambda_{RP} N_{SR}) \\
\frac{dN_{SS}}{dt} &= N_{SS}[(1-s)\lambda_P - \gamma T - A] + \beta(N_{SS} + N_{RS})N_{S\emptyset} \\
\frac{dN_{SR}}{dt} &= N_{SR}[(1-s)\lambda_{RP} - \gamma T - A - \rho\lambda_{\mathbf{PR}}] + \beta(N_{SR} + N_{RR})N_{S\emptyset} \\
\frac{dN_{R\emptyset}}{dt} &= N_{R\emptyset}[\lambda_R - \beta(N_{SS} + N_{SR} + N_{RS} + N_{RR}) - \gamma T - A] + s(\lambda_{RP} N_{RS} + \lambda_{RRP} N_{RR}) \\
\frac{dN_{RS}}{dt} &= N_{RS}[(1-s)\lambda_{RP} - \gamma T - A - \rho\lambda_{\mathbf{PR}}] + \beta(N_{SS} + N_{RS})N_{R\emptyset} \\
\frac{dN_{RR}}{dt} &= N_{RR}[(1-s)\lambda_{RRP} - \gamma T] + \beta(N_{SR} + N_{RR})N_{R\emptyset} + \rho\lambda_{\mathbf{PR}}(\mathbf{N_{RS}} + \mathbf{N_{SR}})
\end{aligned}
\tag{4}
$$

Inclusion of gene transfer between the plasmid and chromosome allows the two forms of resistance to coexist, with the frequency of the low frequency form increasing with the rate of transposition (SI Figure 8). This is analogous to mutation-selection balance. As shown in SI Figure 8, the eventual outcome remains dependent on the initial conditions, with increasing transposition rate increasing the range of initial conditions leading to chromosomal resistance. At very high rates of transposition (10^{-2} per transposon per generation), this leads to chromosomal resistance being the eventual outcome even when the initial frequency of plasmid-borne resistance is very high. However, such high rates are implausible: rate estimates for *Escherichia coli* are of the order of 10^{-5} events per generation per transposon for replicative transposition (and 10^{-8} for non-replicative transposition) [Sousa et al., main text reference 29].

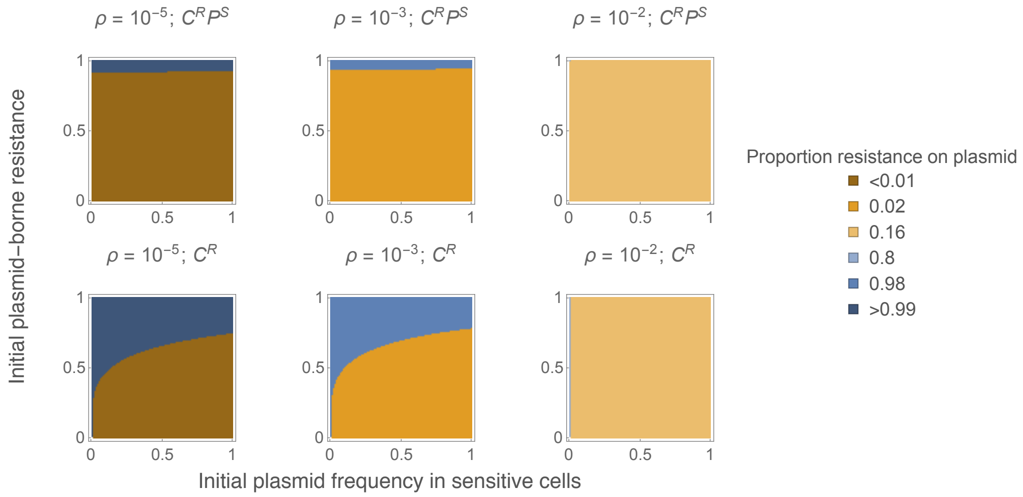

Figure 8: Effect of gene flow between plasmid and chromosome. The panels illustrates the proportion of resistance that is plasmid-borne at equilibrium ($(N_{SR} + N_{RR})/(N_{R\emptyset} + N_{SR} + 2N_{RR})$). ρ is the transposition rate per cell per generation (for *Escherichia coli*, estimates of this rate are of the order of 10^{-5} [Sousa et al., main text reference 29]. Similarly to main text Figure 3, the x-axis indicates the frequency of the sensitive plasmid in the initial sensitive population $N_{SS}/(N_{S\emptyset}+N_{SS})$. The y-axis indicates the frequency of the plasmid-borne resistance in the initial resistant population $N_{SR}/(N_{SR} + N_{C^R C^S})$ for the top panel, $N_{SR}/(N_{SR} + N_{R\emptyset})$ for bottom panel). The total densities of the initial sensitive and resistant populations are both 1. Parameter values are: $\lambda = 1$, $\gamma = 1$, $s = 0.005$, $c_P = 0.075$, $\beta = 0.2$, $A = 1$ and $c_R = 0.05$.

2.5 Imperfect exclusion of plasmid co-infection

In the main text, we assume that carrying one form of the plasmid prevents infection with the other. As such exclusion may not be fully effective, we test the effect of relaxing this assumption. For simplicity, we do not model the co-infected state explicitly: we assume that upon division of a co-infected cell, each daughter cell inherits only one of the plasmids, with equal probability of inheriting either (i.e. half of co-infections result in the super-infecting plasmid replacing the resident plasmid). With k indicating the probability of transferring a plasmid to a cell already carrying a plasmid (compared to a plasmid-free host cell), imperfect exclusion can be approximated as:

$$
\begin{aligned}
\frac{dN_{S\emptyset}}{dt} &= N_{S\emptyset}[\lambda - \beta(N_{SS} + N_{SR} + N_{RS} + N_{RR}) - \gamma T - A] + s(\lambda_P N_{SS} + \lambda_{RP} N_{SR}) \\
\frac{dN_{SS}}{dt} &= N_{SS}[(1-s)\lambda_P - \gamma T - A] + \beta(N_{SS} + N_{RS})(N_{S\emptyset} + \frac{\mathbf{k}}{\mathbf{2}}\mathbf{N_{SR}}) - \frac{\beta\mathbf{k}}{\mathbf{2}}\mathbf{N_{SS}}(\mathbf{N_{SR}} + \mathbf{N_{RR}}) \\
\frac{dN_{SR}}{dt} &= N_{SR}[(1-s)\lambda_{RP} - \gamma T - A + \beta(N_{SR} + N_{RR})(N_{S\emptyset} + \frac{\mathbf{k}}{\mathbf{2}}\mathbf{N_{SR}}) - \frac{\beta\mathbf{k}}{\mathbf{2}}\mathbf{N_{SR}}(\mathbf{N_{SS}} + \mathbf{N_{RS}}) \\
\frac{dN_{R\emptyset}}{dt} &= N_{R\emptyset}[\lambda_R - \beta(N_{SS} + N_{SR} + N_{RS} + N_{RR}) - \gamma T - A] + s(\lambda_{RP} N_{RS} + \lambda_{RRP} N_{RR}) \\
\frac{dN_{RS}}{dt} &= N_{RS}[(1-s)\lambda_{RP} - \gamma T - A] + \beta(N_{SS} + N_{RS})(N_{R\emptyset} + \frac{\mathbf{k}}{\mathbf{2}}\mathbf{N_{RS}}) - \frac{\beta\mathbf{k}}{\mathbf{2}}\mathbf{N_{RS}}(\mathbf{N_{SR}} + \mathbf{N_{RR}}) \\
\frac{dN_{RR}}{dt} &= N_{RR}[(1-s)\lambda_{RRP} - \gamma T] + \beta(N_{SR} + N_{RR})(N_{R\emptyset} + \frac{\mathbf{k}}{\mathbf{2}}\mathbf{N_{RS}}) - \frac{\beta\mathbf{k}}{\mathbf{2}}\mathbf{N_{RR}}(\mathbf{N_{SS}} + \mathbf{N_{RS}})
\end{aligned}
\tag{5}
$$

This change has no impact on the evolutionary stability of plasmid-borne and chromosomal resistance. However, within the region of bi-stability, increasing k increases the range of initial conditions in which the outcome is plasmid-borne resistance (SI Figure 9). This is because co-infection increases the advantage the resistant plasmid acquires from plasmid transmission, thus allowing it to increase in frequency more rapidly even in presence of the sensitive plasmid.

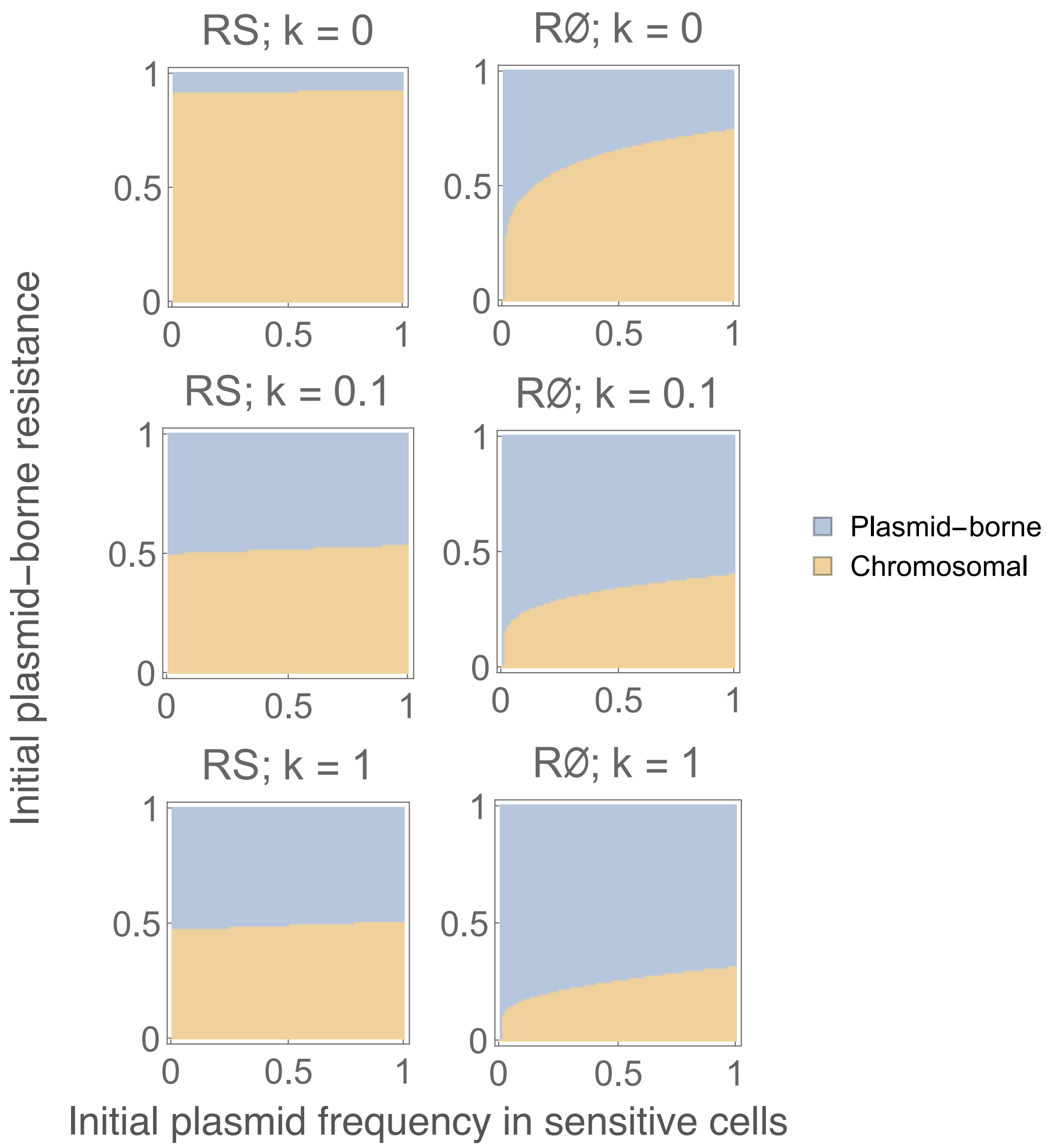

Figure 9: Effect of initial conditions on equilibrium location of the resistance gene, with imperfect exclusion of plasmid co-infection. As in main text Figure 3, the panels illustrates whether plasmid-borne (blue) or chromosomal resistance (orange) are observed at equilibrium. The x-axis indicates the frequency of the sensitive plasmid in the initial sensitive population. The y-axis indicates the frequency of the plasmid-borne resistance in the initial resistant population. The initial resistant population consists either of RS and SR cells (left) or $R\emptyset$ and SR cells (right). The rows correspond to increasing probability of co-infection (k). Parameter values are: $\lambda = 1$, $\gamma = 1$, $s = 0.005$, $c_P = 0.075$, $\beta = 0.2$, $A = 1$ and $c_R = 0.05$.

2.6 Temporally fluctuating selection

In the main text, we treat the antibiotic-associated death rate as a constant. In natural settings, selection pressure from antibiotics is often heterogeneous, either through spatial (see 'Local Adaptation' section below) or temporal variation in antibiotic concentration. We therefore modify the model to include a fluctuating antibiotic-associated death rate. The model structure remains identical to that presented in the main text, but with antibiotic-associated death rate modelled as: $A[1 + sin(2\pi/T)]$. That is, a sine wave with period T and mean and amplitude A.

Because of the switch to fluctuating selection, we can no longer apply the linear stability analysis approach used in the main text to determine the evolutionary stability of plasmid-borne and chromosomal resistance in this system. We therefore use simulation to check whether established plasmid-borne resistance can be displaced by introduction of chromosomal resistance at low frequency, and vice versa.

As illustrated in SI Figure 10, the presence of bi-stability is robust to the inclusion of fluctuation. Fluctuation favours plasmid-borne resistance, but the effect is dependent on the period of the fluctuation.

This relationship arises because the relative fitness of chromosomal and plasmid-borne resistance is dependent on both the relative frequencies of the two forms of resistance and the frequency of the sensitive plasmid. These frequencies are affected differently by phases of positive and negative selection. Thus, the relationship between the characteristics of fluctuating selection (i.e. phase, amplitude, period) and which resistance is favoured is likely to be complex.

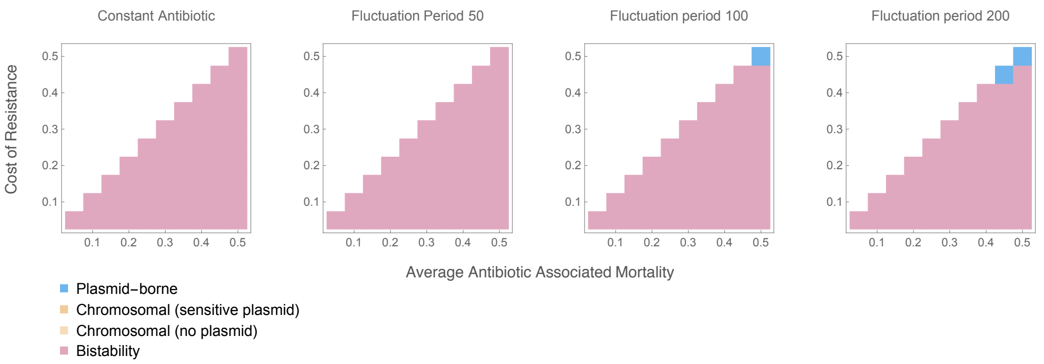

Figure 10: The effect of fluctuating selection on the evolutionary stability of chromosomal and plasmid-borne resistance: the presence of bi-stability is robust, with plasmid-borne resistance favoured under some conditions. Each simulation is started from the equilibrium reached when only one form of resistance (plasmid-borne or chromosomal) is included in the model. The other form is then introduced at low frequency (10^{-4}). If the low frequency form of resistance is able to increase in frequency and invade, the established resistance is not evolutionarily stable. Parameter values are: $\lambda = 1$, $\gamma = 1$, $s = 0.005$, $c_P = 0.075$, $\beta = 0.2$, $A = 1$ and $c_R = 0.05$.

3 Local adaptation

We modify the model in the main text to include influx of cells from a sensitive population. Here, μ is the overall rate of influx of sensitive cells, and q is the proportion of the incoming cells which carry the sensitive plasmid. In our simulations, the initial population is fully resistant (and comprised of SR and RS cells in varying proportions). The modified dynamics are given by:

$$\begin{aligned}
\frac{dN_{S\emptyset}}{dt} &= N_{S\emptyset}[\lambda - \beta(N_{SS} + N_{SR} + N_{RS} + N_{RR}) - \gamma T - A] + s(\lambda_P N_{SS} + \lambda_{RP} N_{SR}) + (\mathbf{1} - \mathbf{q})\mu \\
\frac{dN_{SS}}{dt} &= N_{SS}[(1-s)\lambda_P - \gamma T - A] + \beta(N_{SS} + N_{RS})N_{S\emptyset} + \mathbf{q}\mu \\
\frac{dN_{SR}}{dt} &= N_{SR}[(1-s)\lambda_{RP} - \gamma T - A + \beta(N_{SR} + N_{RR})N_{S\emptyset} \\
\frac{dN_{R\emptyset}}{dt} &= N_{R\emptyset}[\lambda_R - \beta(N_{SS} + N_{SR} + N_{RS} + N_{RR}) - \gamma T - A] + s(\lambda_{RP} N_{RS} + \lambda_{RRP} N_{RR}) \\
\frac{dN_{RS}}{dt} &= N_{RS}[(1-s)\lambda_{RP} - \gamma T - A] + \beta(N_{SS} + N_{RS})N_{R\emptyset} \\
\frac{dN_{RR}}{dt} &= N_{RR}[(1-s)\lambda_{RRP} - \gamma T] + \beta(N_{SR} + N_{RR})N_{R\emptyset}
\end{aligned} \tag{6}$$

The effect of this modification depends on the frequency of the sensitive plasmid in the incoming cells (q): an influx of sensitive cells carrying the sensitive plasmid favours chromosomal resistance whereas an influx of sensitive cells without the plasmid favours plasmid-borne resistance (see the main text).

4 Plasmid persistence

We consider the relationship between plasmid transmissibility and the location of resistance genes in more detail. This relationship is relevant to the question of plasmid persistence: under the low transmissibility assumption (i.e. plasmids are not transmissible enough to persist as parasites), Bergstrom et al. show that beneficial genes will always locate on the chromosome rather than plasmid (in absence of local adaptation) [main text reference 16]. Thus, the persistence of low transmissibility plasmids is a paradox: they cannot be maintained without beneficial genes, but beneficial genes cannot be maintained on these plasmids. Here, it is worth noting that Bergstrom et al. refer specifically to persistence of a non-beneficial plasmid in a host population where the chromosome also lacks the beneficial gene.

In our framing, this corresponds to persistence of the sensitive plasmid in a chromosomally sensitive population.

To check whether this result also holds in our model structure, we use linear stability analysis to compare the parameter space in which plasmid-borne resistance is evolutionarily stable with the parameter space in which parasitic plasmids are viable. In other words, we compare the space in which resistance genes can locate on the plasmid (despite competition from chromosomal resistance) with the space in which a sensitive plasmid can be maintained in a chromosomally sensitive population (in absence of competition from the resistant plasmid). Contrary to Bergstrom et al., we find that resistance genes *can* locate onto the plasmid even when plasmid transmissibility is too low for parasitic plasmids to be viable (SI Figure 11). Thus, low transmissibility plasmids can theoretically persist because of the advantage they provide their host cells.

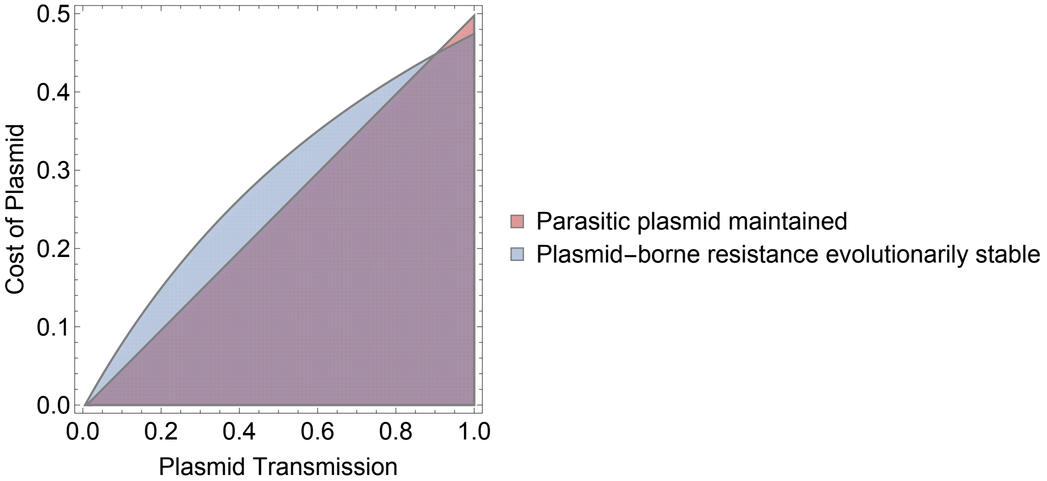

Figure 11: Resistance can locate onto the plasmid even when plasmid transmissibility is low, allowing low transmissibility plasmids to persist because of the advantage they provide their host cells. The plot shows the region of evolutionary stability of plasmid-borne resistance, i.e. where resistance can occur on the plasmid despite competition from chromosomal resistance (blue); the region where a parasitic plasmid (i.e. a sensitive version of the plasmid in a sensitive population) can persist (red); and the overlap of these regions (purple). Note that the blue region in which plasmid-borne resistance is evolutionarily stable is bi-stable: the resistance gene is not necessarily on the plasmid; which form of resistance is observed at equilibrium depends on the initial frequencies. Values for non-varying parameters are the same as in main text, except antibiotic associated mortality is lower (i.e. resistance is beneficial, but not essential, otherwise the sensitive cell population would not persist at all): $\lambda = 1$, $\gamma = 1$, $s = 0.005$, $c_P = 0.075$, $\beta = 0.2$, $A = 0.5$ and $c_R = 0.05$.

5 Additional Figures

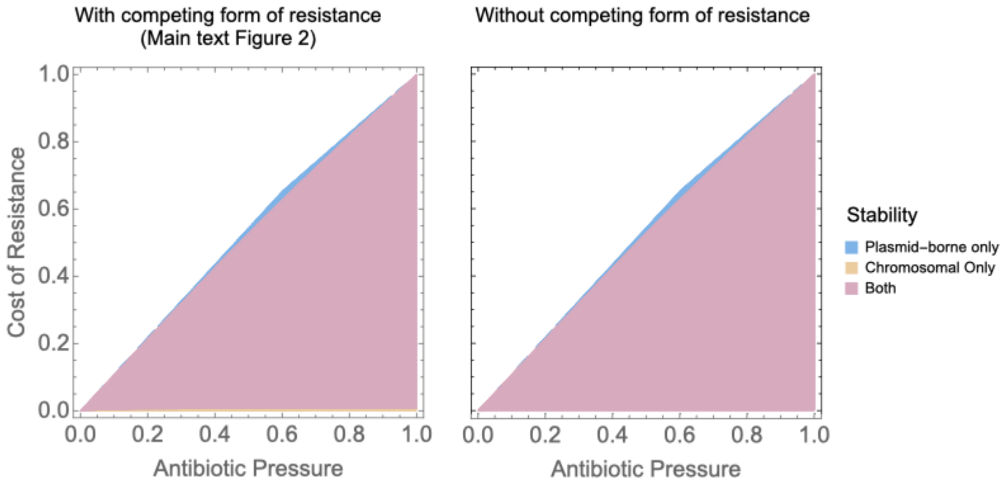

Figure 12: Stability of plasmid-borne and chromosomal resistance in absence of competition from the other resistance form (but in presence of the sensitive chromosome and sensitive plasmid).The left-hand panel shows the evolutionary stability depicted in main text Figure 2. The right-hand panel shows stability in absence of competition from the other resistance form. For chromosomal resistance, these two areas are the same. For plasmid-borne resistance, there is a region at low fitness cost (narrow orange band at the bottom of the left-hand panel) where plasmid-borne resistance is stable in absence of competition from chromosomal resistance, but not evolutionary stable i.e. not stable in presence of competition from chromosomal resistance. Parameter values are $\lambda = 1$, $\gamma = 1$, $s = 0.005$, $c_P = 0.075$, $\beta = 0.2$, $A = 1$ and $c_R = 0.05$.

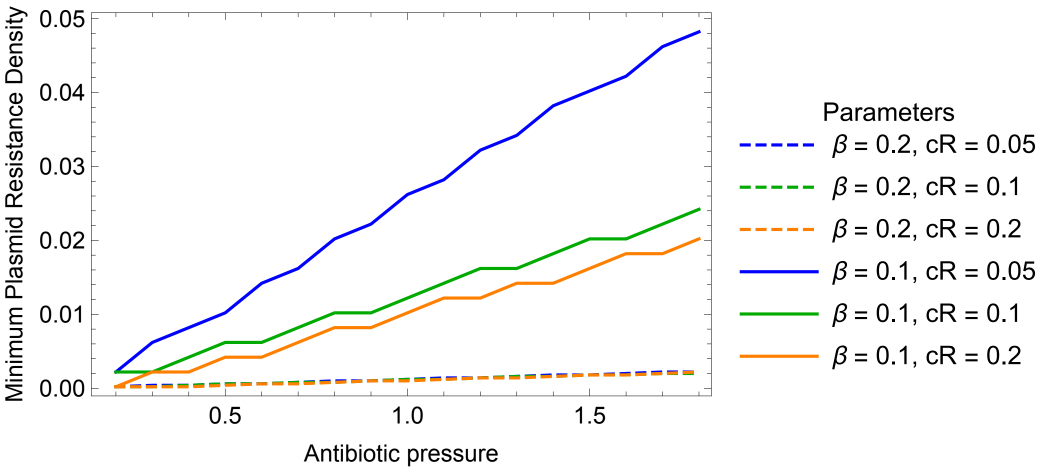

Figure 13: Low densities of plasmid-borne resistance are enough to prevent invasion by chromosomal resistance. The plot shows the minimum initial density of plasmid-borne resistance (N_{SR}) for which chromosomal resistance introduced at low density ($N_{RS} = 10^{-4}$) cannot invade. The initial conditions for other cell types are $N_{S\emptyset} = 0.2$ and $N_{SS} = 0.70$ (approximating equilibrium in absence of antibiotics when $\beta = 0.1$), and 0 for all others. Parameters are $\lambda = 1$, $\gamma = 1$, $s = 0.005$, $c_P = 0.075$.

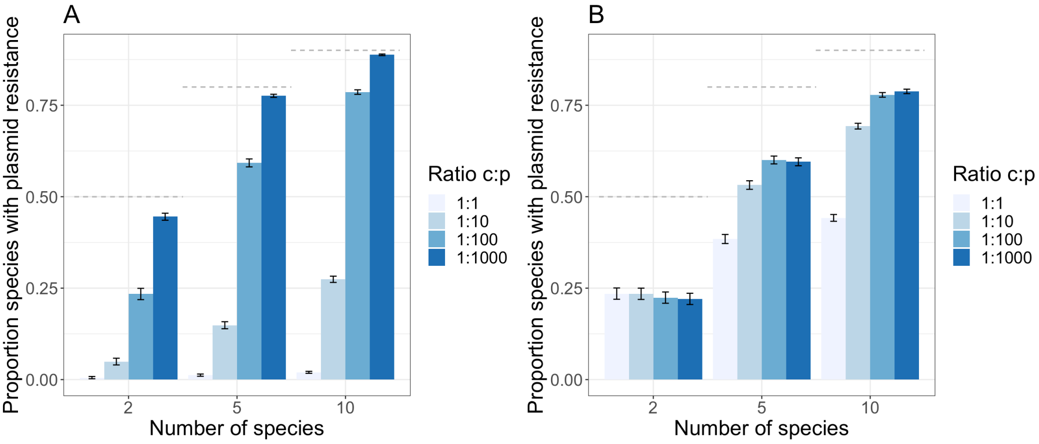

Figure 14: Spread of resistance genes between species for rate parameters differing from those used to generate Figure 5 in the main text. As in the main text figure, the bars indicate the proportion of plasmid-borne resistance depending on the number of simulated species and the ratio of the rate of interspecies transfer of the chromosomal (c) and plasmid-borne gene (p). The horizontal lines show the maximum proportion of plasmid resistance, given that resistance must first emerge on the chromosome ($(n-1)/n$). Error bars represent 95% confidence intervals based on 1000 realisations. A: With a lower rate of gene flow between gene and chromosome than in the main text ($t = 10^{-2}$ instead of $t = 10^{-1}$ in the main text, $m = 10^{-6}$ and $c = 10^{-5}$ as in the main text). The lower rate of gene flow means chromosomal resistance has more time to spread before a plasmid-borne resistance arises, leading to a lower proportion of species with plasmid borne resistance. B: Here, we assume the rate of acquiring plasmid-borne resistance from a species with chromosomal resistance is independent of the rate of interspecies plasmid transfer, and similarly, that the rate of acquiring chromosomal resistance from a species with plasmid-borne resistance is independent of the rate of interspecies transfer of chromosomal genes. In this simulation, both these rates are 10^{-5} and $m = 10^{-6}$ and $c = 10^{-5}$ as in the main text. The effect of variation in p is reduced compared to the main text because the the rate at which plasmid-borne resistance arises does not depend on p.
